# The Effect of Fe Addition in the RM(Nb)IC Alloy Nb–30Ti–10Si–2Al–5Cr–3Fe–5Sn–2Hf (at.%) on Its Microstructure, Complex Concentrated and High Entropy Phases, Pest Oxidation, Strength and Contamination with Oxygen, and a Comparison with Other RM(Nb)ICs, Refractory Complex Concentrated Alloys (RCCAs) and Refractory High Entropy Alloys (RHEAs)

**DOI:** 10.3390/ma15175815

**Published:** 2022-08-23

**Authors:** Nikos Vellios, Panos Tsakiropoulos

**Affiliations:** Department of Materials Science and Engineering, Sir Robert Hadfield Building, The University of Sheffield, Mappin Street, Sheffield S1 3JD, UK

**Keywords:** Nb-silicide-based alloys, refractory metal intermetallic composites (RMICs), refractory high entropy alloys (RHEAs), refractory complex concentrated alloys (RCCAs), pest oxidation, complex concentrated phases, high entropy phases, compositionally complex phases

## Abstract

In this work, the RM(Nb)IC alloy Nb–30Ti–10Si–5Cr–5Sn–3Fe–2Al–2Hf (NV2) was studied in the as-cast and heat-treated conditions; its isothermal oxidation at 700, 800 and 900 °C and its room temperature hardness and specific strength were compared with other Sn-containing RM(Nb)ICs—in particular, the alloy Nb–24Ti–18Si–5Cr–5Fe–5Sn (NV5)—and with RCCAs and RHEAs. The addition of Fe (a) stabilised Nb_ss_; A15–Nb_3_X (X = Al, Si and Sn) and Nb_3_Si; metastable Nb_3_Si-m’ and Nb_5_Si_3_ silicides; (b) supported the formation of eutectic Nb_ss_ + Nb_5_Si_3_; (c) suppressed pest oxidation at all three temperatures and (d) stabilised a Cr- and Fe-rich phase instead of a C14–Nb(Cr,Fe)_2_ Laves phase. Complex concentrated (or compositionally complex) and/or high entropy phases co-existed with “conventional” phases in all conditions and after oxidation at 800 °C. In NV2, the macrosegregation of Si decreased but liquation occurred at T >1200 °C. A solid solution free of Si and rich in Cr and Ti was stable after the heat treatments. The relationships between solutes in the various phases, between solutes and alloy parameters and between alloy hardness or specific strength and the alloy parameters were established (parameters δ, Δχ and VEC). The oxidation of NV2 at 700 °C was better than the other Sn-containing RM(Nb)ICs with/without Fe addition, even better than RM(Nb)IC alloys with lower vol.% Nb_ss_. At 800 °C, the mass change of NV2 was slightly higher than that of NV5, and at 900 °C, both alloys showed scale spallation. At 800 °C, both alloys formed a more or less continuous layer of A15–Nb_3_X below the oxide scale, but in NV5, this compound was Sn-rich and severely oxidised. At 800 °C, in the diffusion zone (DZ) and the bulk of NV2, Nb_ss_ was more severely contaminated with oxygen than Nb_5_Si_3_, and the contamination of A15–Nb_3_X was in-between these phases. The contamination of all three phases was more severe in the DZ. The contamination of all three phases in the bulk of NV5 was more severe compared with NV2. The specific strength of NV2 was comparable with that of RCCAs and RHEAs, and its oxidation at all three temperatures was significantly better than RHEAs and RCCAs.

## 1. Introduction

The most challenging application in aero-turbine engines with the greatest potential benefit from new ultra-high temperature materials (UHTMs) are high-pressure turbines (HPT). In this application, the key material properties for HPT blades are creep strength, oxidation resistance, ultimate strength and fracture toughness [1,2]. Metallic UHTMs with load-carrying capabilities beyond those of Ni-based superalloys at high temperatures are under development and include refractory metal intermetallic composites (RMICs), refractory metal high entropy alloys (RHEAs) and refractory metal complex concentrated alloys (RCCAs) [3,4,5,6,7].

RMICs are based on Nb (i.e., RM(Nb)ICs) or Mo (i.e., RM(Mo)ICs). Some of the former are RHEAs or RCCAs, i.e., RM(Nb)ICs/RHEAs or RM(Nb)ICs/RCCAs [5,6]. RM(Nb)ICs are also known as Nb in situ composites or Nb-silicide-based alloys or Nb in situ silicide composites [8,9]. Currently, single crystal internally cooled and coated Ni-based superalloys in HPT applications provide a maximum surface temperature capability of about 1150 °C. RM(Nb)ICs have melting temperatures in excess of 1800 °C and, with environmental coatings [3,7], would allow a substantial increase in the surface temperature. 

RHEAs and RCCAs share the same alloying elements with RM(Nb)ICs [4,5,6]. The RM(Nb)ICs are multiphase materials, with key phases: the bcc Nb solid solution (Nb_ss_) and Nb_5_Si_3_ silicide, and additional (secondary) phases: Nb_3_Si silicide(s); A15–Nb_3_X (X = Al, Ge, Si and Sn) compounds and the C14–NbCr_2_ Laves phase. The volume fraction and distribution of these phases within RM(Nb)ICs are important for their properties [9,10]. RHEAs and RCCAs can be single-phase or multiphase alloys with key phases: bcc solid solution(s) with/without M_5_Si_3_ silicide(s), and/or Laves phase(s), where M = transition metal (TM) or refractory metal (RM) [4]. However, often, it is not stated if there are different types of silicides or Laves phases. In RM(Nb)ICs and RM(Nb)ICs/RCCAs, RM(Nb)ICs/RHEAs complex concentrated (CC) phases (also referred to as compositionally complex (CC) phases) or high entropy (HE) phases can form (coexist) and/or be stable together with “conventional” phases [7,9].

The stability of the aforementioned phases in RM(Nb)ICs and RM(Nb)ICs/RCCAs or RM(Nb)ICs/RHEAs and the type of Nb_5_Si_3_ (meaning tetragonal α or β Nb_5_Si_3_ and hexagonal γNb_5_Si_3_ [11]) are crucial for the performance of these alloys and depend on alloying additions and temperature. The alloying elements can be grouped based on their effectiveness (a) in low and high temperature strength and creep, fracture toughness, oxidation behaviour in the range of temperatures of pest oxidation and at high temperatures and (b) in controlling the vol.% and type of bcc solid solution(s), and the vol.% of A15–Nb_3_X and C14–NbCr_2_ Laves phases [5,6,7,9,12]. Aluminium, Cr, Hf, Si and Ti are indispensable alloying additions and must be used together with one or two of the three elements B, Ge and Sn, which play a key role in oxidation, plus TMs and RMs to get a balance of the mechanical properties with oxidation resistance [7]. To date, at least 23 elements have been used in RM(Nb)ICs and RM(Nb)ICs/RCCAs or RM(Nb)ICs/RHEAs and 12 in RHEAs and RCCAs, albeit not all in the same metallic UHTM [4,5,6]. 

In RM(Nb)ICs and RM(Nb)ICs/RCCAs or RM(Nb)ICs/RHEAs, extrinsic toughening is provided by the bcc Nb_ss_. The silicides have no intrinsic ductility. The creep of Nb_ss_ is inferior to that of the aforementioned intermetallics [9,13]. Furthermore, the tetragonal (tP32) Nb_3_Si and the hexagonal (hP16) γNb_5_Si_3_ have inferior creep properties compared with the tetragonal (tI32) Nb_5_Si_3_ [13]. The most promising properties for binary (unalloyed) A15–Nb_3_Al and C15–NbCr_2_ were toughness, creep strength and ultimate strength for the former and oxidation and creep strength of the latter, with the minimum creep rate (s^−1^) at 1200 °C and 172 MPa, respectively, 7.2 × 10^−6^ s^−1^ and 4.5 × 10^−6^ s^−1^ [14], compared with 1 × 10^−8^ s^−1^ for binary (i.e., unalloyed) Nb_5_Si_3_ [13]. The oxidation of Nb_3_Si is inferior to that of Nb_5_Si_3_.

tP32 Nb_3_Si is useful to engineer microstructures via the phase transformations L → Nb_ss_ + Nb_3_Si and Nb_3_Si → Nb_ss_ + αNb_5_Si_3_. The stability of Nb_3_Si can be affected by alloying additions, for example, Al [15] or Sn [16], whereas other elements, for example, Cr, promote the eutectoid transformation and the C14–NbCr_2_ Laves phase [15]. Iron is believed to favour the C14–Nb(Cr,Fe)_2_ Laves phase in RM(Nb)ICs [8]. Of particular interest for the work reported in this paper is the synergy of Sn and Fe with Al, Cr, Hf, Si and Ti in RM(Nb)ICs and the stability of Nb_3_Si. Let us expand on these points.

Alloying with Fe resulted in a tensile elongation of 2% and 95%, respectively, at room temperature and at 1300 °C and superplasticity above 1350 °C, with tensile elongation at 512% reported at 1450 °C for the Nb–16Si–2Fe alloy [17]. In the Nb–10Si–2Fe alloy, (a) the as-sintered microstructure consisted of the Nb_ss_, Nb_3_Si, Nb_5_Si_3_ and Nb_4_Fe_3_Si_5_ phases, and after annealing, Nb_3_Si transformed into Nb_ss_ and αNb_5_Si_3_; (b) the fracture toughness increased from K_Q_ = 11.2 MPa√m for the as-sintered condition to K_Q_ = 20.1 MPa√m for the extruded and heat-treated condition and (c) Nb_ss_ and Nb_4_Fe_3_Si_5_ were suggested to play an important role in the fracture process—in particular, the latter [18]. The microstructure of mechanically alloyed and plasma spheroidized powder particles of Nb–25Ti–16Si–2Al–2Cr–2Fe RM(Nb)IC consisted of the Nb_ss_, Nb_3_Si and Nb_5_Si_3_ phases [19]. 

Iron forms hP16 Fe_5_Si_3_ (prototype Mn_5_Si_3_, the same as for the metastable hP16 γNb_5_Si_3_ [11]). According to Raghavan, in the Fe–Nb–Si ternary system at 1150 °C, the tetragonal αNb_5_Si_3_ can be in equilibrium with the C14–NbFe_2_ Laves (ε phase) or with Fe_2_Nb_3_ or with ε and Fe_2_Nb_3_ or with Fe_2_Nb_3_ and FeNb_4_Si (τ_4_ phase). Additionally, there is three-phase equilibria between the ε phase, Fe_7_Nb_6_ (μ phase) and Fe_2_Nb_3_, and αNb_5_Si_3_ is not in equilibrium with Nb_4_Fe_3_Si_5_ (τ_3_ phase) [20]. In the same ternary system, the Fe_2_Nb_3_ phase was not confirmed at 1000, 1100 and 1200 °C by Wang et al., according to which, αNb_5_Si_3_ can be in equilibrium with the ε phase or with the μ phase or with the ε and μ phases or with the μ and τ_4_ phases, and the ε phase can be in equilibrium with the μ phase [21]. Wang et al. reported large Si solubilities in the ε and μ phases (respectively, ≤27.7 and ≤14.7 at.%), confirmed that there is no equilibrium between αNb_5_Si_3_ and τ_3_ and showed a small solubility of Fe (≤3.1 at.%) in the silicide. 

Nb_ss_ and αNb_5_Si_3_ were the equilibrium phases in the RM(Nb)IC alloy Nb–24Ti–18Si–5Cr (KZ4) at 1500 °C, in which the addition of Cr had promoted the formation of the C14–NbCr_2_ Laves phase in the cast alloy and enhanced the eutectoid transformation Nb_3_Si → Nb_ss_ + αNb_5_Si_3_ during solidification [15]. When Fe was added in the RM(Nb)IC alloy Nb–24Ti–18Si–5Cr–5Fe (NV3 [22]), the cast microstructure consisted of Nb_ss_, Nb_5_Si_3_, tP32 Nb_3_Si and the metastable Nb_3_Si-m and Nb_3_Si-m’ silicides [11], FeNb_4_Si (τ_4_) and Ti-rich oxide; the aforementioned eutectoid transformation was retarded, and the sensitivity of the formation of Nb_3_Si to solidification conditions increased. After heat treatment (1200 °C/100 h), the same phases were present as in NV3-AC, with the exception of tP32 Nb_3_Si and the vol.% of the metastable Nb_3_Si-m’ had increased and the compositions of both metastable silicides had shifted closer to the stoichiometric composition. In other words, Fe in Sn-free RM(Nb)ICs stabilised 3-1 silicides (stable and metastable), and instead of a C14 Laves phase, other compounds such as the τ_3_ or τ_4_ intermetallics were formed.

Opinions about the role of Fe in the oxidation of RM(Nb)ICs differ. According to Bewlay et al., alloying with Fe improved oxidation owing to the formation of the C14–Nb(Cr,Fe)_2_ Laves phase [8], but then, according to Menon et al., “the replacement of Cr with Fe may have a detrimental effect in oxidation resistance” [23]. We attribute this difference of opinion (a) to the fact that the oxidation of multicomponent RM(Nb)ICs is affected by the addition of B, Ge or Sn and the synergy of these elements with Al, Cr, Hf and Ti [7,9,24,25,26,27,28,29] and (b) to the chemical compositions of the studied RM(Nb)ICs, where Fe was in synergy with Al, Cr, Ge, Hf, Si, Sn and Ti in the case of Bewlay et al. (they studied RM(Nb)ICs with a low Si content (12.5 at.%), Ti = 22.5 at.% and as-cast microstructures consisting of Nb_ss_ and Nb_5_Si_3_) or Fe was in synergy with Al, B, Ce, Cr, Ge, Hf, Si, Sn and Ti in the case of Menon et al. (they studied RM(Nb)ICs with low and high Si contents (12.6–17.6 at.% Si), 24.8 < Ti < 26.2 at.%, and the heat-treated microstructures consisted of Nb_ss_, Nb_5_Si_3_ and Ti_5_Si_3_), which would not have made it possible for both groups of researchers to infer how Fe performed in their multicomponent multiphase RM(Nb)ICs.

Table 1 summarises the data about the phases that were stable after heat treatment in systematically studied RM(Nb)ICs with/without Sn or Fe addition. The data shows (a) that the stability of Nb_ss_ can be affected by the synergy of (i) Al, Sn and Ti (alloy ZX6); (ii) Fe, Sn and Ti (alloy NV8); (iii) Al, Hf, Sn and Ti (alloy EZ5); (iv) Al, Cr, Hf, Sn and Ti (alloy EZ8) and (v) Cr, Fe, Sn and Ti (alloy NV5); (b) that the synergy of Fe, Sn and Ti can (i) destabilise Nb_5_Si_3_ (alloy NV8) and (ii) stabilise Nb_3_Si (alloy NV8); (c) that A15–Nb_3_X can be stable even with a 2 at.% Sn addition in alloys, where (i) Al, Sn and Ti (alloy ZX5) or (ii) A, Cr, Sn and Ti (alloy ZX7) are in synergy; (d) that Nb_3_Si is stabilised by the synergy of Fe, Sn and Ti (alloy NV8); (e) that metastable Nb_3_Si-m is stabilised with the synergy of Cr, Fe, and Ti (alloy NV3); (f) that metastable Nb_3_Si-m’ is stabilised with the synergy (i) of Cr, Fe, and Ti (alloy NV3) and (ii) of Cr, Fe, Sn and Ti (alloy NV5) and (g) that in Fe-containing RM(Nb)ICs with/without Sn addition, the FeNb_4_Si (τ_4_) is stable instead of the C14 Laves phase.

In other words, regarding 3-1 silicide and A15–Nb_3_X, (i) the synergy of Cr with Fe stabilised metastable 3-1 silicides instead of the tP32 Nb_3_Si (alloy NV3), (ii) the synergy of Fe with Sn stabilised tP32 Nb_3_Si and A15–Nb_3_Sn (alloy NV8) and (iii) the synergy of Cr and Fe with Sn stabilised A15–Nb_3_Sn and metastable Nb_3_Si-m’ (alloy NV5), and the stability of the bcc Nb_ss_ was “controlled” with the additions of Al and Sn (alloys ZX6, EZ5 and EZ8) or with the addition of Fe and Sn (alloys NV8 and NV5).

The motivation for the research presented in this paper was to study how the synergy of Fe and Sn with Al, Cr, Hf and Ti affected (a) the stability of Nb_ss_ and A15–Nb_3_X (X = Al, Si and Sn) and the Nb_3_Si and Nb_5_Si_3_ silicides; (b) oxidation in the pest temperature range; (c) the formation of eutectic Nb_ss_ + Nb_5_Si_3_ and (d) the formation of the C14–Nb(Cr,Fe)_2_ Laves phase. We selected the RM(Nb)IC alloy 43Nb–30Ti–10Si–2Al–5Cr–3Fe–5Sn–2Hf (alloy NV2, nominal composition (at.%) rounded to the nearest whole number, calculated composition 42.6Nb–30.3Ti–10.2Si–2.1Al–4.9Cr–3.1Fe–4.9Sn–1.9Hf), which was designed using the alloy design methodology NICE [9]. There was not enough data for Fe in the NICE database; thus, for the calculation of the alloy composition, Fe was calculated using the Cr database. The calculations gave Al(2.1) + Cr(8) + Sn(4.9) = 15 at.%, where in each parenthesis is given the calculated concentration of each element. The Cr and Sn concentrations in NV2 were set based on the data for the Fe-containing alloys NV3, NV5 [22] and NV8 [31], where the concentrations of Cr and Sn were equal, and the Fe concentration was the balance with 15 at.%. For the design of the alloy, the property target was mass change 5 mg/cm^2^ for isothermal oxidation at 800 °C for 100 h. The constraints of the alloy design were as follows: The RM(Nb)IC (i) must contain Al, Fe, Hf and Sn; (ii) should have a room temperature hardness higher than 600 HV and (iii) should have a higher vol.% A15–Nb_3_X (X = Al, Si and Sn) than Nb_ss_. The property target was informed by the results of the NV1 alloy [32]. The constraint (i) was chosen because of (a) and (b); for the constraint (ii), we were advised by the results of the NV5 alloy [22] and the alloys EZ5, EZ6 and EZ8 [30], and the constraint (iii) was preferred owing to the results of alloys EZ6 and EZ8 [30] (for nominal alloy compositions, see Table 1).

The structure of the paper is as follows. After the experimental details, in the Results section, the microstructure of the alloy in the as-cast and heat-treated conditions is described separately, data is given for the density and alloy hardness and, then, the results for the oxidation of the alloy in three pest oxidation temperatures are presented together with data for the oxidation of alloy NV5 with which NV2 is compared in the discussion. The latter revisits the alloy design, briefly discusses the macrosegregation in NV2 and, then, the characteristic features of the phases are considered—in particular, drawing attention to the solid solution in the heat-treated alloy, followed by the discussion of oxidation and hardness from the perspective of the alloy design methodology NICE, and then, a comparison is made with RCCAs. Contamination of the phases with oxygen from below the oxide scale/substrate interface to the bulk of the alloys is considered to highlight how alloying can hinder the contamination of the phases. The last part focuses on the CC or HE phases in the cast, heat-treated and oxidised alloy.

## 2. Experimental

High purity elements (Nb (99.99 wt%), Si (99.999 wt%), Hf (99.99 wt%), Ti (99.99 wt%), Cr (99.9 wt%), Al (99.9 wt%), Fe (99.98 wt%) and Sn (99.99 wt%)) were used as the starting materials to prepare ingots of 300 g in weight using clean melting with a water-cooled copper crucible. Specimens that were cut from the bulk of the ingots were wrapped in Ta foil and heat-treated at 1000 °C or 1200 °C in a tube furnace for 100 h under a Ti-gettered argon atmosphere. The heat treatment temperatures were decided owing to the liquation observed at higher temperatures. Note that the alloys NV3, NV5 and NV8 were also not heat-treated above 1200 °C [22,31]. In NV2, the liquation was attributed to the higher concentration of Ti and the addition of Fe and Sn. 

The as-cast (AC) and heat-treated (HT) alloys were studied using X-ray diffraction (XRD) and Electron Probe Microanalysis (EPMA). A Philips X-ray diffractometer with a monochromatic Cu Kα (λ = 1.5418) radiation was used for the identification of the phases, which was done using JCPDS data. Backscatter electron (BSE) imaging and quantitative chemical analyses were performed using a JEOL 8600 EPMA. High purity elements Hf_2_Si [33] and Al_2_O_3_ that had been polished to a 1-μm finish were used for standardisation purposes. At least ten analyses were done for each phase in different parts (top, bulk and bottom) of the ingot. The average, standard deviation and the minimum and maximum concentrations of each element are given in the tables in the paper. Area fractions of Nb_ss_ and A15–Nb_3_X were measured for the same areas that were used for large area (×350) analyses in EPMA using the software available on the microprobe instrument. At least ten measurements were taken for each phase or large area.

The isothermal oxidation of the alloy was studied at 700, 800 and 900 °C for up to 100 h using a Stanton Redcroft thermobalance equipped with an alumina tube furnace. Small cube-shaped specimens (5 × 5 × 5 mm^3^) were cut from the as-cast ingot. Cross-sections of oxidised specimens were studied using EPMA. The Vickers hardness (HV) of the AC or HT alloy was measured with a load of 10 kg. At least 10 measurements were taken for each condition. The density of the alloys was measured using the Archimedes’ principle and a Sartorius LA2305 electronic precision balance, equipped with a density determination kit. The average, standard deviation and the minimum and maximum values of the hardness and density of the alloys are given in this paper.

## 3. Results

### 3.1. As-cast (NV2–AC)

**Figure 1:**
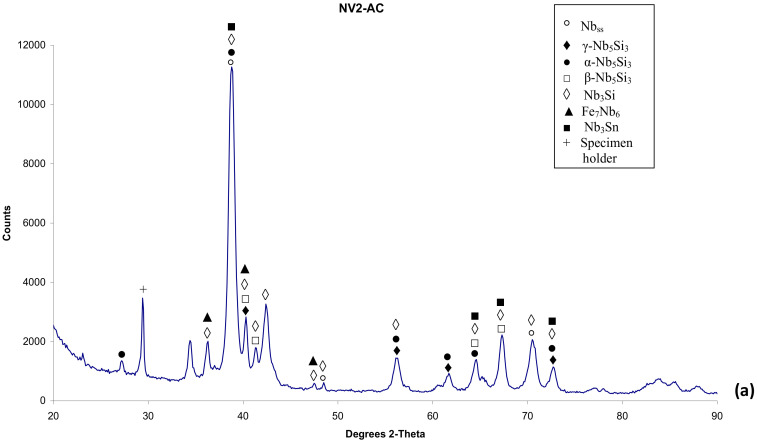

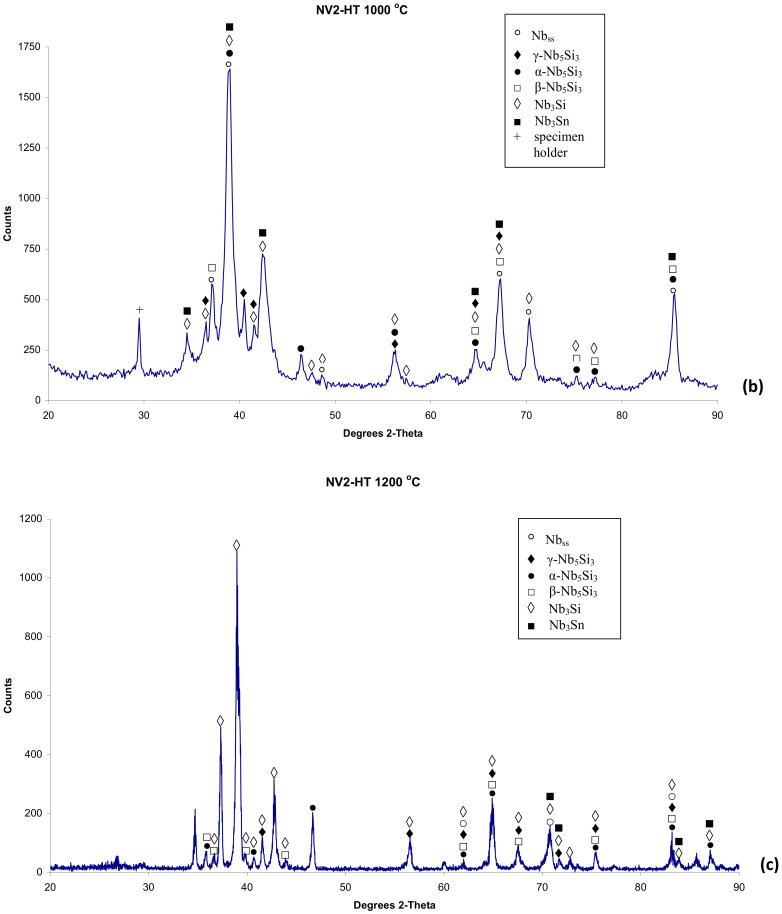
X-ray diffractograms of (**a**) NV2–AC, (**b**) NV2–HT 1000 °C and (**c**) NV2–HT 1200 °C.

The density of the alloy was 6.97 g/cm^3^ (Table 2). The actual composition was 42.2Nb–30.2Ti–10.3Si–2.3Al–4.9Cr–2.9Fe–5.2Sn–2Hf, and there was a macrosegregation of Si (MACSi = C_max_^Si^ − C_min_^Si^ = 2.5 at.% Si [34]). According to the XRD data (Figure 1a), tetragonal α and β Nb_5_Si_3_ and hexagonal γNb_5_Si_3_ were present in the microstructure, together with Nb_3_Si, Nb_3_Sn and Fe_7_Nb_6_ (μ phase). According to the chemical analysis data (Table 3), the phases present in NV2–AC were Nb_ss_; A15–Nb_3_X (X = Al, Si and Sn); Nb_5_Si_3_; Nb_3_Si; metastable Nb_3_Si-m’ and the μ phase. There was Ti-rich Nb_ss_ and Ti and Hf-rich Nb_5_Si_3_. There was also eutectic Nb_ss_ and Nb_5_Si_3_. The aforementioned phases were not observed in all parts of the ingot. Note that Table 3 gives the average compositions of the phases in the whole ingot.

**Figure 2:**
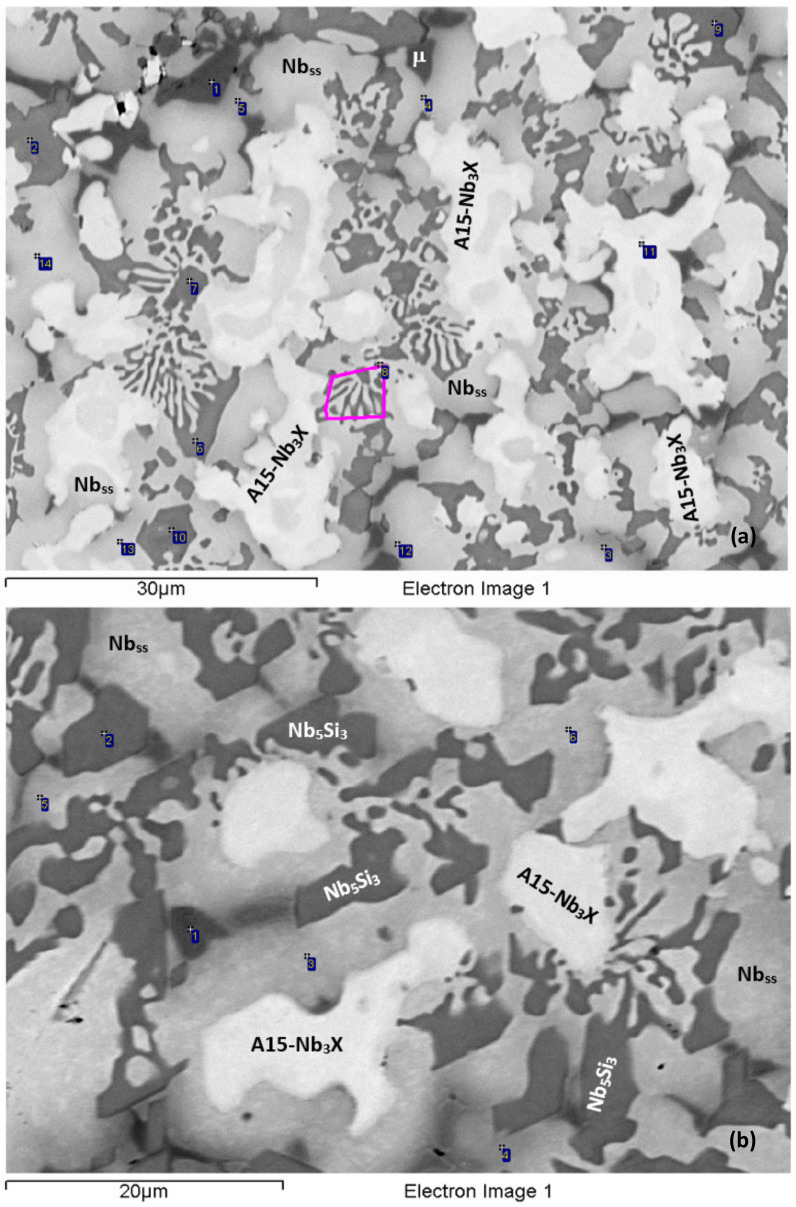

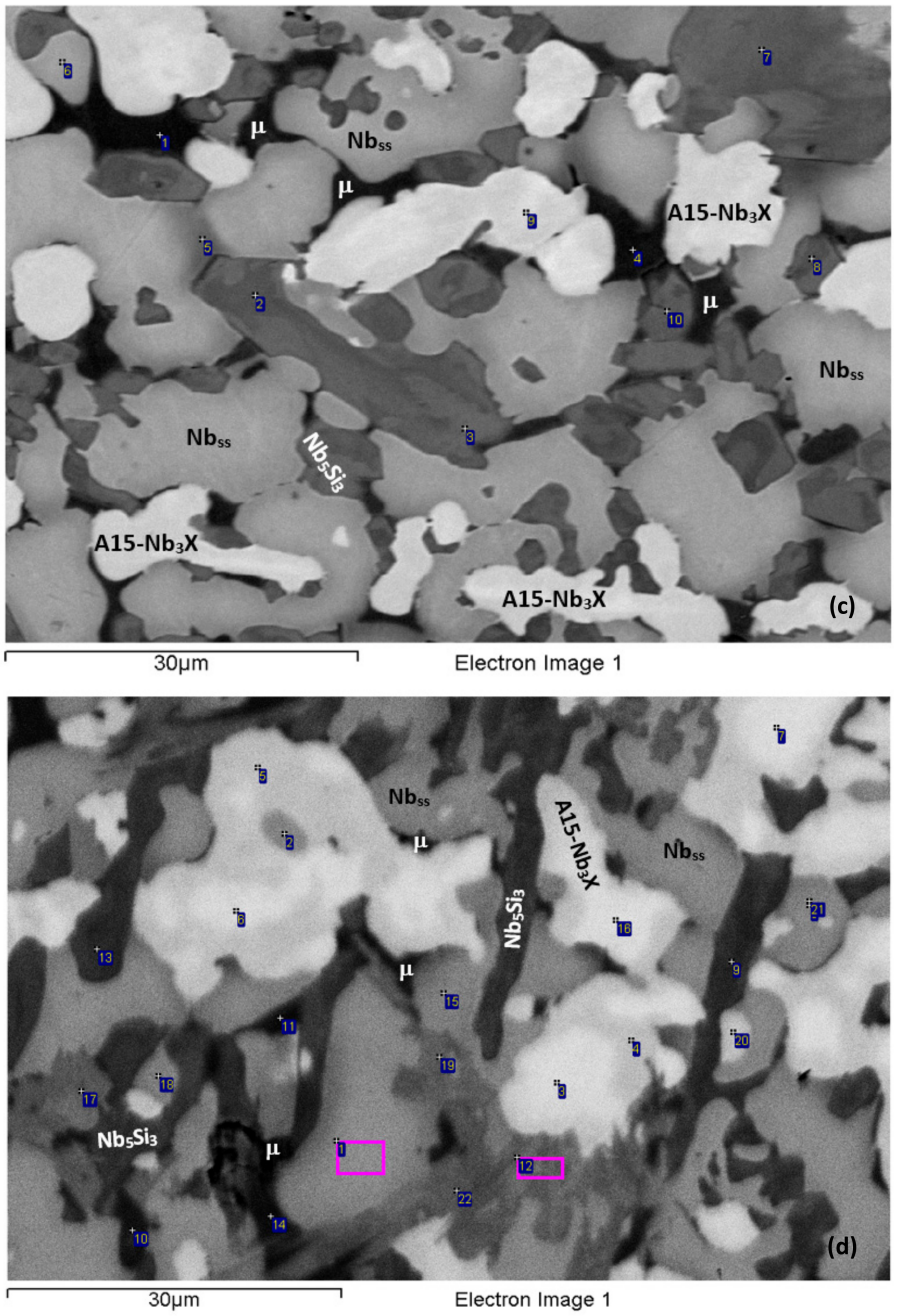
BSE images of the microstructure of NV2–AC: (**a**) top, (**b**) bulk (**c**) and (**d**) bottom of the ingot. In (**a**), 1 = Fe_7_Nb_6_ (μ-phase); 2, 6, 7, 9 and 10 = Nb_5_Si_3_; 13 and 14 = Nb_ss_ and 3, 4, 5 and 12 = Ti-rich Nb_ss_, where, for 3, 5 and 12 Nb/Ti < 1, 11 = A15–Nb_3_X, where a slightly darker contrast indicates a lower Sn concentration, and8 = (Nb_ss_ + Nb_5_Si_3_)_eutectic_; (**b**) 1 = Fe_7_Nb_6_, 2 = Nb_5_Si_3_, 3, 5, 6 = Nb_ss_ and 4 = Ti-rich Nb_ss_ with Nb/Ti < 1; (**c**) 1 and 4 = Fe_7_Nb_6_; 3 and 10 = Nb_5_Si_3_; 2, 7 and 8 = Ti-rich Nb_5_Si_3_; 5 = Ti-rich Nb_ss_ (Nb/Ti < 1); 6 = Nb_ss_ and 9 = A15–Nb_3_X and (**d**) 1, 2, 15, 18 and 21 = Nb_ss_; 3, 4, 5, 6, 7, 16 and 20 = A15–Nb_3_X; 9 and 13 = Nb_5_Si_3_; 10, 11 and 14 = Fe_7_Nb_6_; 12 = Nb_3_Si and 19 and 22 = Nb_3_Si-m’.

Near the top of the ingot, the Nb_ss_, A15–Nb_3_X, Nb_5_Si_3_ and Fe_7_Nb_6_ phases were observed (Figure 2a). Nb_5_Si_3_ had Nb/(Ti + Hf) = 0.83 and Si + Al + Sn = 35.9 at.%, and the highest analysed concentrations of Ti and Hf, respectively, were 30.8 and 4.8 at.%. The silicide corresponded to hexagonal γNb_5_Si_3_ owing to its Nb/(Ti + Hf) ratio [33]. The Ti-rich Nb_ss_ had Nb/Ti > 1, and its highest analysed Ti concentration was 37 at.%. Ti-rich Nb_ss_ was mainly formed adjacent to Fe_7_Nb_6_ or A15–Nb_3_X. The Nb_ss_ + Nb_5_Si_3_ eutectic formed in areas very close to the surface of the ingot. The average composition of the eutectic was 39.9Nb–30.8Ti–16.5Si–2Al–3.6Cr–1.8Fe–2.7Sn–2.7Hf. The μ phase was observed either at the interface of A15–Nb_3_X with Nb_ss_ or between A15–Nb_3_X grains or adjacent to Nb_5_Si_3_. The μ phase was rich in Cr. 

In the bulk of the ingot (Figure 2b), the microstructure was coarser and consisted of the same phases as in the top. The eutectic formed at a significantly lower vol.%. Three types of Nb_5_Si_3_ silicide were observed owing to their chemical compositions. The first was Ti and Hf-rich Nb_5_Si_3_ with Nb/(Ti + Hf) = 0.79 and Si + Al + Sn = 36.9 at.%, in which the highest analysed Ti and Hf concentrations, respectively, were 30.7 and 7.4 at.% and corresponded to hexagonal γNb_5_Si_3_ owing to its Nb/(Ti + Hf) ratio being less than 1 [33]. The second was Ti, Al and Cr-poor and Fe-free Nb_5_Si_3_ with Nb/(Ti + Hf) = 1.84 and Si + Al + Sn = 37.3 at.%, in which the highest Ti and Hf concentrations, respectively, were 19.8 and 2.9 at.%, had formed at a low vol.% and corresponded to tetragonal Nb_5_Si_3_. The third was Nb_5_Si_3_ with Si + Al + Sn = 34.4 at.% and Nb/(Ti + Hf) = 1.38, in which the highest Ti and Hf concentrations, respectively, were 26.6 and 2.9 at.%. The latter Nb_5_Si_3_ was also tetragonal but was richer in Fe and Sn than the former two. The Ti-rich Nb_ss_ was even richer in Ti, and its Ti concentration was as high as 50 at.% (Nb/Ti < 1) in some parts in the bulk of the ingot. A15–Nb_3_X had a similar composition as in the top but formed at a lower vol.% compared with the top of the ingot. The μ phase formed at a small vol.% and was slightly richer in Cr compared with the top of the ingot.

The phases that were present in the bulk of the ingot also were found in the bottom together with Nb_3_Si and metastable Nb_3_Si-m’ (Figure 2c,d). Additionally, there was the Nb_ss_ + Nb_5_Si_3_ eutectic. Nb_3_Si formed at a very low vol.%. Nb_3_Si-m’ was found next to A15–Nb_3_X or at the interface of the latter with Nb_ss_. The solubility of Fe in Nb_3_Si was low and increased slightly in Nb_3_Si-m’. The solubilities of Al, Cr and Sn in Nb_3_Si were higher than in Nb_3_Si-m’. The concentration of Hf was essentially the same in both 3-1 silicides. The solubility range of Ti in Nb_3_Si was wider compared with Nb_3_Si-m’: 23.3–31.5 at.% in the former and 21.3–22.8 at.% in the latter. The average composition of the eutectic in the parts of the bottom where only A15–Nb_3_X and the eutectic were present, sometimes with a few very small grains of the μ phase, was 40Nb–31.1Ti–15.2Si–1.9Al–3.8Cr–2.9Fe–3.8Sn–1.8Hf, whereas, in the parts of the bottom where all the aforementioned phases were present, the composition of the eutectic was 37.5Nb–31.1Ti–18.9Si–1.7Al–2.8Cr–2.8Fe–2.8Sn–2.4Hf. The eutectic in the bottom of the ingot was richer in Fe compared with the top.

### 3.2. Heat-Treated (NV2–HT)

**Figure 3:**
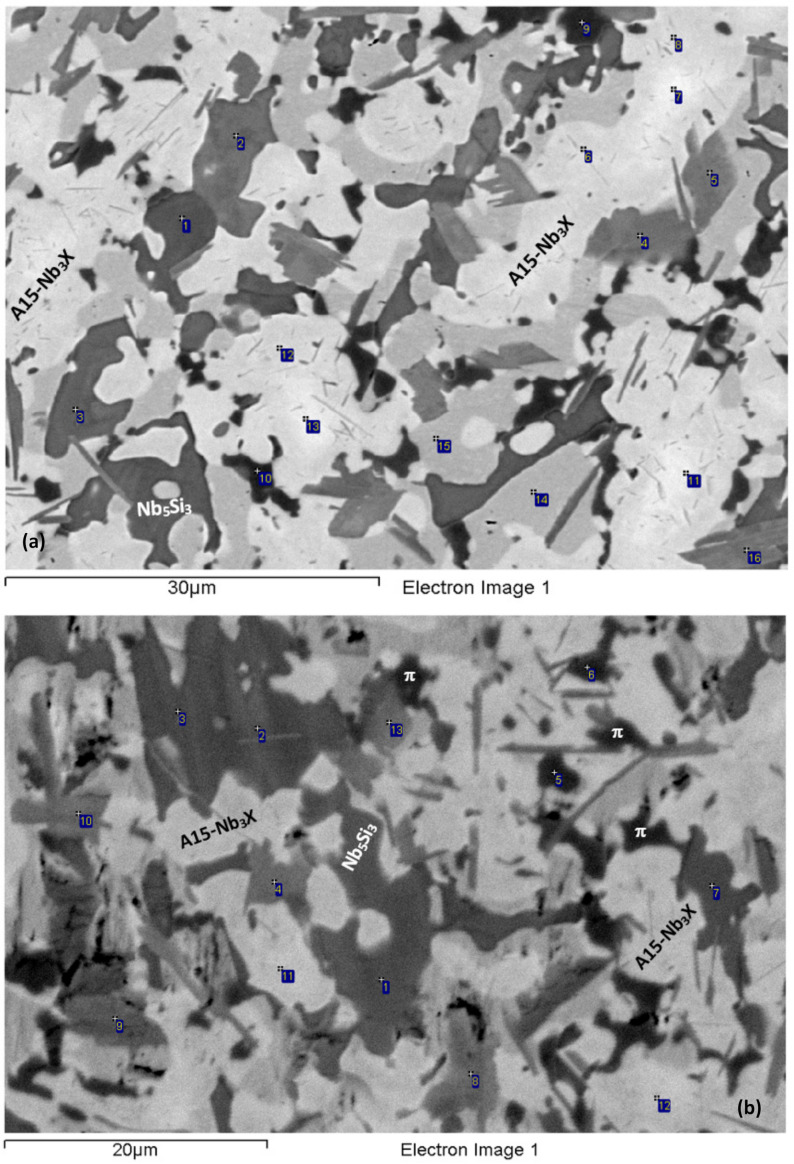

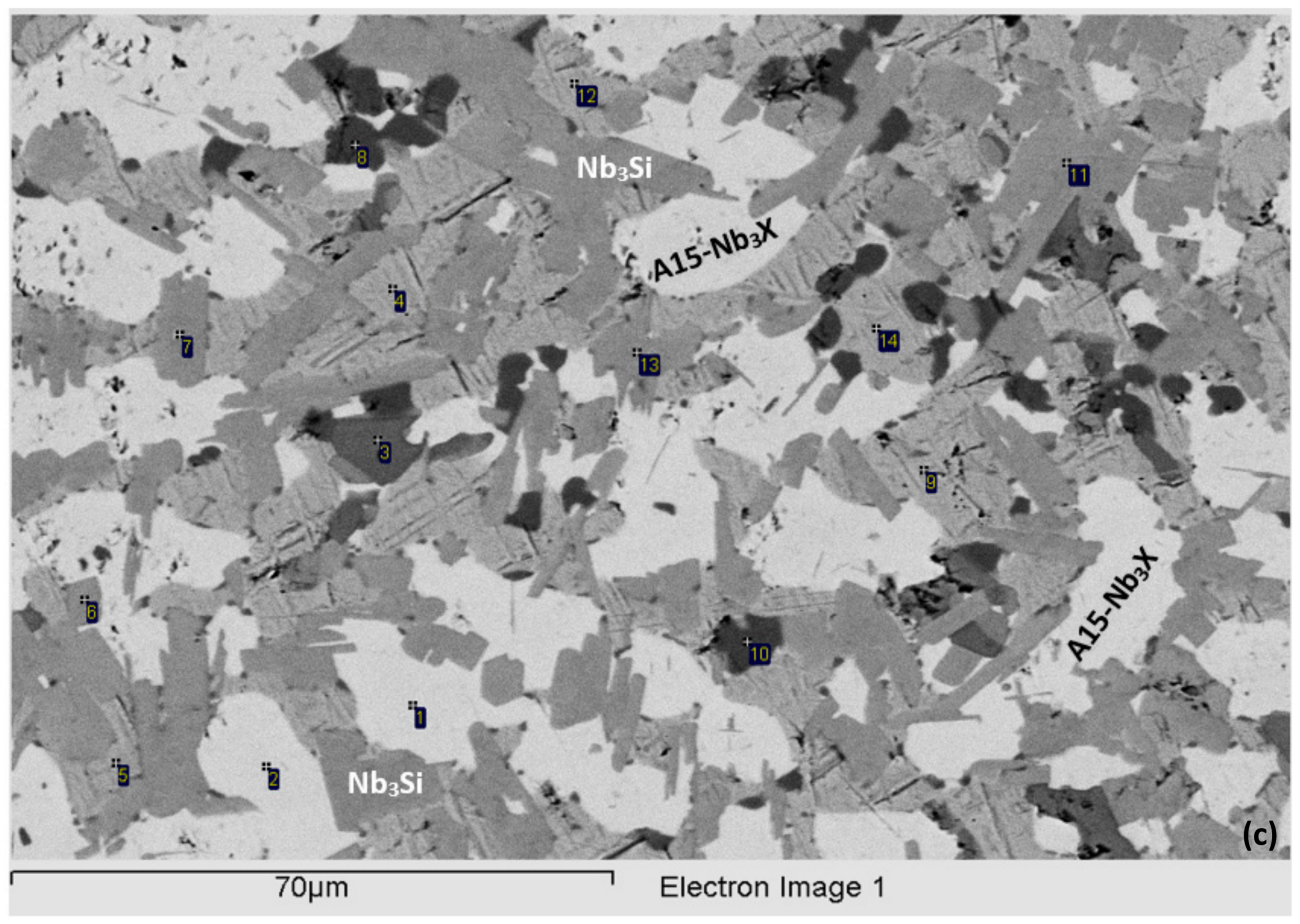
BSE images of the bulk of NV2–HT. (**a**,**b**) HT1 = 1000 °C/100 h and (**c**) HT2 = 1200 °C/100 h. In (**c**), the contrast has been enhanced. In (**a**) 1, 2 and 3 = Nb_5_Si_3_; 4, 5 and 16 = Nb_3_Si-m’; 6, 7, 8, 11, 12 and 13 = A15–Nb_3_X; 14 and 15 = Nb_ss_ with no Si and 10 = the π phase; (**b**) 1, 2, 3 and 7 = Nb_5_Si_3_; 4, 8 and 10 = Nb_3_Si-m’; 5 and 6 = the π phase; 9 and 13 = Nb_3_Si and 11 and 12 = A15–Nb_3_X and (**c**) 1 and 2 = A15–Nb_3_X; 3 = Nb_5_Si_3_; 4, 5, 9, 12 and 14 = Nb_ss_ with no Si; 7 = Nb_3_Si; 6, 11 and 13 = Nb_3_Si-m’ and 8 and 10 = the π phase.

The alloy was heat-treated at two temperatures, namely at 1000 °C and 1200 °C (see Section 2). The actual composition of the alloy after the heat treatment at 1000 °C for 100 h (NV2–HT1) was 40.9Nb–30.5Ti–10.9Si–2.2Al–5Cr–3.1Fe–5.2Sn–2.2Hf. The NV2–HT1 was a complex concentrated alloy, i.e., RM(Nb)IC/RCCA. The typical microstructure is shown in Figure 3a,b. Compared with the cast alloy, the vol.% of Nb_ss_ and A15–Nb_3_X, respectively, decreased and increased (Table 2). A15–Nb_3_X was richer in Ti and poorer in Si than NV2–AC. According to the XRD data (Figure 1b), tetragonal α and β Nb_5_Si_3_ and hexagonal γNb_5_Si_3_ were present. Some of the Nb_5_Si_3_ grains were poor in Si and richer in Cr and Fe, with Si + Al + Sn = 33.9 at.%, compared with 36.6 at.% for the “normal” Nb_5_Si_3_ that was poor in Cr and Fe (Table 3). Both Nb_5_Si_3_ types had Nb/(Ti + Hf) about 0.75 that corresponds to hexagonal γNb_5_Si_3_, according to [33]. 

Extensive studies using XRD did not provide evidence for the C14–NbCr_2_ Laves phase or the C14–NbFe_2_ Laves phase after this heat treatment and the one at 1200 °C. Additionally, Fe_7_Nb_6_ was not confirmed with XRD and was not confirmed after detailed studies of the heat-treated microstructures using EPMA. The EPMA data gave a phase that was richer in Cr than Fe_7_Nb_6_, similarly with NV5–AC [22], which we defined as the π phase (Table 3) based on the results for NV5–AC [22]. The π phase was observed where Fe_7_Nb_6_ was in NV2–AC. Compared with Fe_7_Nb_6_, the Cr concentration in the π phase was higher both in NV5–AC [22] and NV2–HT. In NV2–HT, the Si, Fe, Ti and Al concentrations in the π phase were slightly lower. In NV2, in the π and μ phases. the solubility of Sn and Hf were similar (Table 3). Additionally, tP32 Nb_3_Si and Nb_3_Si-m’ were observed, the latter with a higher vol.%. The 3-1 silicide was found adjacent to the Nb_5_Si_3_ or the π phase. Compared with NV2–AC, the composition of Nb_3_Si-m’ was closer to that of tP32 Nb_3_Si. 

After the heat treatment at 1200 °C for 100 h (NV2-HT2), the actual composition was 41.4Nb–31.1Ti–11.1Si–2Al–4.9Cr–3.2Fe–5.1Sn–2.2Hf. The typical microstructure is shown in Figure 3c and consists of Nb_ss_, A15–Nb_3_X, Hf-rich Nb_5_Si_3_, the π phase, Nb_3_Si and Nb_3_Si-m’. The vol.% of Nb_ss_ and A15–Nb_3_X decreased compared with NV2–HT1 (Table 2). According to the XRD data (Figure 1c), tetragonal α and β Nb_5_Si_3_ and hexagonal γNb_5_Si_3_ were present. The number of peaks in the diffractogram that corresponded to tP32 Nb_3_Si increased. Fe_7_Nb_6_ was not confirmed by XRD and EPMA. A15–Nb_3_X was surrounded either by Nb_ss_ or by Nb_3_Si. Both Nb_ss_ and A15–Nb_3_X became richer in Ti and Al (Table 3). The Sn concentration in A15–Nb_3_X increased. Nb_3_Si was often faceted. Nb_5_Si_3_ was Hf-rich, formed at a low vol.% and its chemical composition was very close to that in NV2–AC. The π phase was present in all parts of the microstructure, often adjacent to A15–Nb_3_X. 

In NV2–HT1 and NV2–HT2, there were many Nb_ss_ grains that were Si-free, i.e., Nb_ss_ with no Si, as well as “normal” Nb_ss_ (Table 3). All the chemical analysis data for Nb_ss_ gave average compositions of 52.5Nb–34.8Ti–0.2Si–2Al–4.5Cr–2.1Fe–1Hf–2.9Sn and 47.2Nb–38Ti–0.1Si–2.7Al–5.1Cr–1.9Fe– 1.2Hf–3.8Sn, respectively, for NV2–HT1 and NV2–HT2.

### 3.3. Hardness

The hardness of the alloy is given in Table 2 for the as-cast and heat-treated conditions. There was a small increase of the hardness after the heat treatment. 

### 3.4. Oxidation

**Figure 4:**
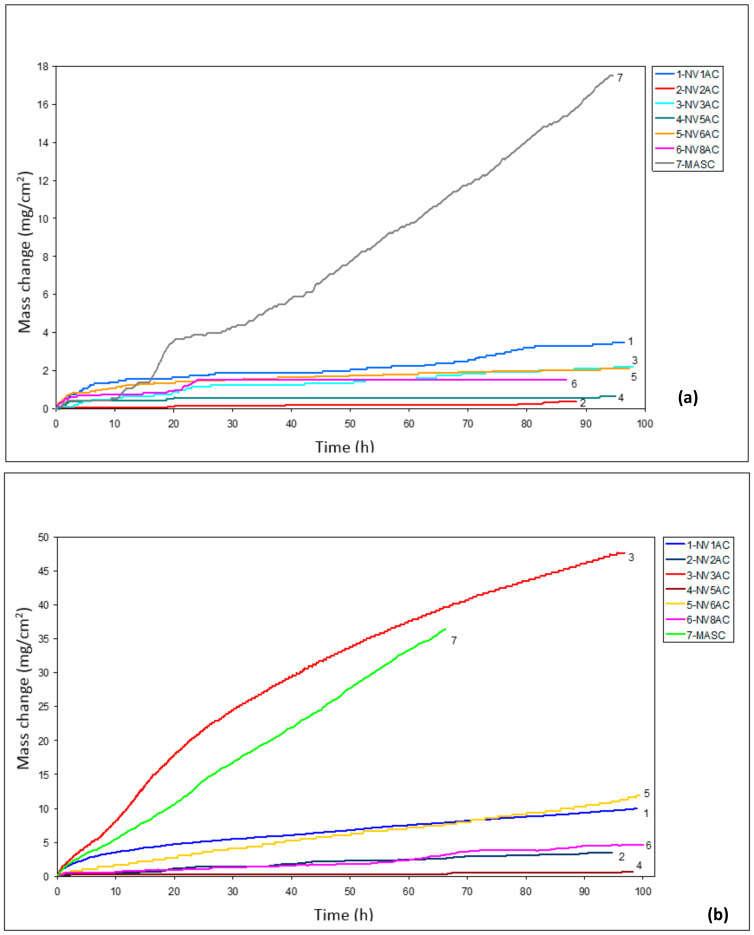

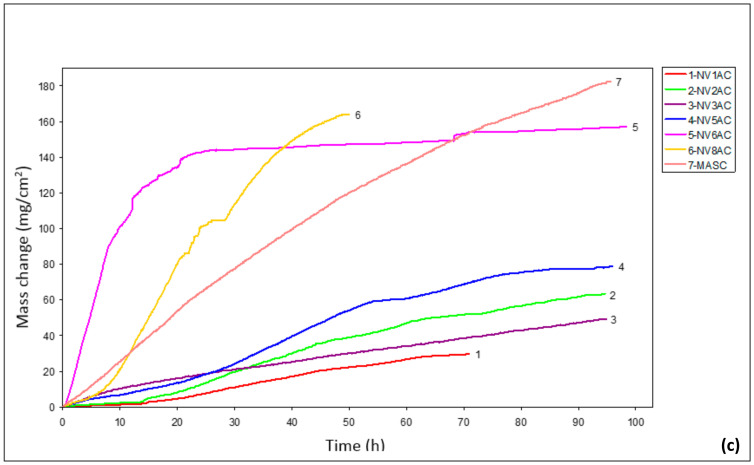
Mass change data for alloys NV1, NV2, NV3, NV5, NV6 and NV8 and the MASC alloy at (**a**) 700 °C, (**b**) 800 °C and (**c**) 900 °C.

**Figure 5:**
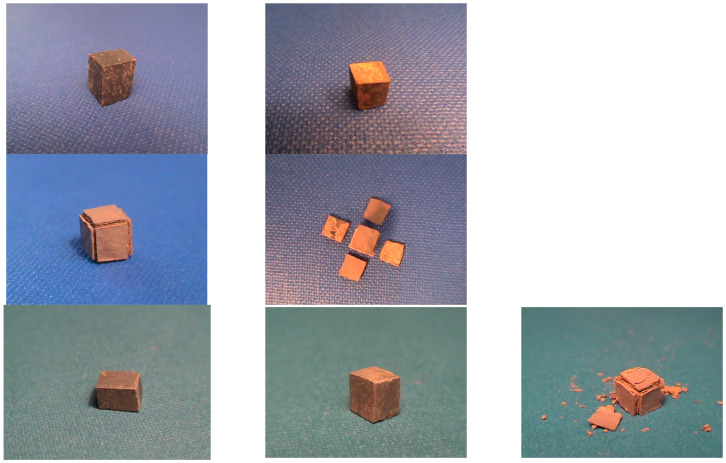
Images of specimens of alloys NV2 and NV5 after isothermal oxidation for 100 h. Top row, alloy NV2, left after oxidation at 700 °C and right at 800 °C. Middle row alloy NV2 after oxidation at 900 °C, left Maltese cross before separation of the oxide scale and right after separation of the oxide scale. Bottom row, alloy NV5, left after oxidation at 700 °C, middle at 800 °C and right at 900 °C.

The isothermal oxidation of the as-cast alloy was studied at 700, 800 and 900 °C. The mass change data is shown in Figure 4, the oxidised specimens in Figure 5 and the oxidation rate constants are given in Table 4. In Figure 4 and Table 4, data for the cast alloys NV1 [32], NV3, NV5, NV6 and NV8 is also included for comparison purposes (for the nominal compositions of the alloys, see Table 1). Note that the alloys NV1 and NV6 were Fe-free. The alloy NV9 (Nb–18Si–5Sn [16]) suffered from pest oxidation at all three temperatures, and thus, it is not included in Figure 4. Alloy NV2 was also compared with the as-cast MASC alloy (Nb–25Ti–16Si–8Hf–2Al–2Cr, [3,8]) at the same temperatures. The alloys in Figure 4 were produced following the same procedure as for the alloy of this study. 

At 700 °C, the mass change of alloy NV2 was the lowest (0.5 mg/cm^2^) (Figure 4a). Alloy NV2 followed parabolic oxidation kinetics, similar to all the other NV series alloys (Table 4). At 800 °C the mass change of alloy NV2 (3.45 mg/cm^2^) was higher than that of NV5 (Figure 4b), and NV2 followed parabolic oxidation kinetics, similar to alloys NV5 and NV8 (Table 4). At 900 °C, the mass change of NV2 (55 mg/cm^2^) was slightly higher than that of NV3 and significantly lower than NV8 (Figure 4c), but as with all the NV series alloys and the MASC alloy, it followed linear oxidation kinetics with similar rate constants (Table 4). 

At 700, 800 and 900 °C, alloy NV2 did not pest, at the former two temperatures, its oxide scale did not spall off and, at 900 °C, it formed a Maltese cross, parts of which spalled off easily upon handling (Figure 5). Alloy NV5 did not pest at 700 and 800 °C and, at 900 °C, also formed a Maltese cross, parts of which spalled off (Figure 5). Alloy NV8 suffered from catastrophic pest oxidation at 900 °C (the specimen was converted to powder), but similarly with alloys NV2 and NV5, it did not pest at the two lower temperatures. Alloy NV6 did not pest at 700 and 800 °C; at the latter temperature, there was evidence of the very early stages of Maltese cross-formation, but at 900 °C, it suffered from catastrophic pest oxidation. 

**Figure 6:**
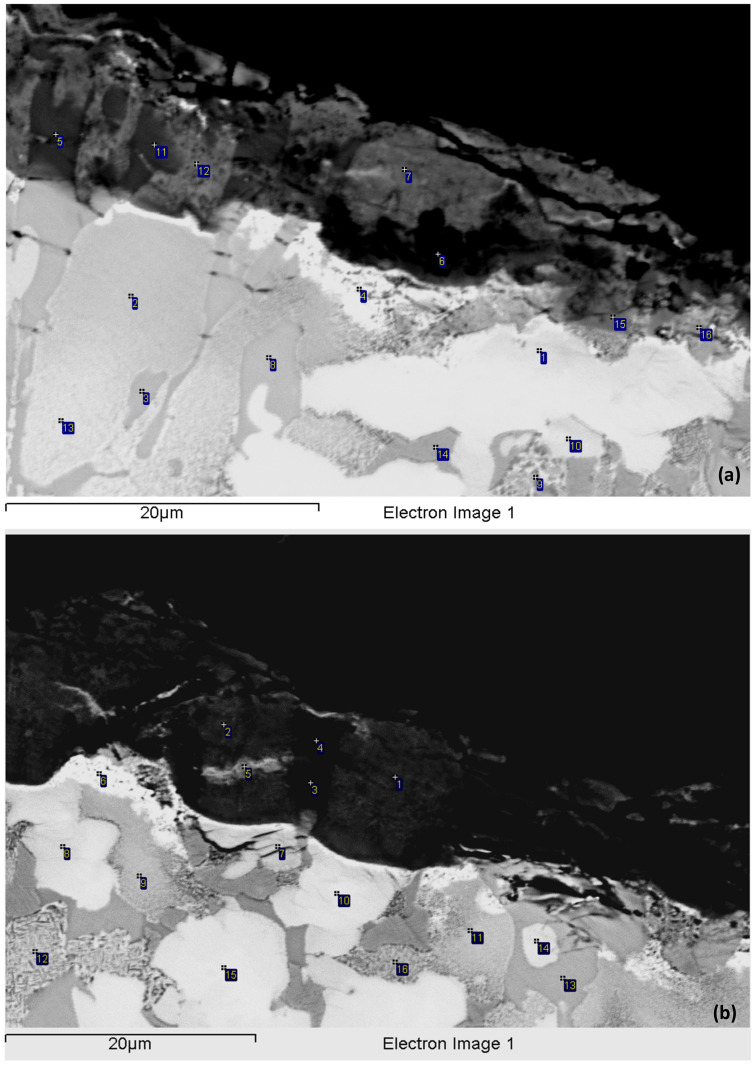
Oxide scale and diffusion zone of NV2 after isothermal oxidation at 800 °C for 100 h. In (**a**), 2 = Nb_ss_, 3 and 8 = Nb_5_Si_3_, 10 = A15–Nb_3_X, 4 = Sn-rich A15–Nb_3_X, 5 and 11 = Si-rich oxide, 7 and 12 = Nb and Ti-rich oxide and 6 = Ti-rich oxide. In (**b**), 1 and 2 = Nb and Ti-rich oxide; 3 and 4 = Ti-rich oxide; 5 = Sn-rich area in scale surrounded by Nb and Ti-rich oxide and Ti-rich oxide; 6 = Sn-rich A15–Nb_3_X; 10, 14 and 15 = A15–Nb_3_X; 12 and 16 = severely oxidised Nb_ss_ and 13 = Nb_5_Si_3_, and the π phase is the grey contrast phase between analyses 10 and 15.

The scale and bulk of the isothermally oxidised specimen of alloy NV2 at 800 °C was studied further and was compared (a) with alloy NV5, owing to the “comparable” oxidation behaviour of both alloys, and (b) with alloy NV1 [32], which had a very high vol.% of Nb_ss_, was Fe-free and its mass change at 900 °C was the lowest (Figure 4c). The oxide scale of NV2 was non-uniform, but its non-uniformity was less pronounced than that of NV1, and the average scale thickness was about 15 μm. Fewer cracks were present in the scale, compared with NV1. At the interface of the oxide scale with A15–Nb_3_X or Nb_ss_ in the substrate, a thick and rather continuous layer of Sn-rich A15–Nb_3_X that exhibited very bright contrast under BSE imaging formed (Figure 6), as was the case in NV1 [32]. This layer was thicker and practically continuous compared with NV1. This layer was not observed in the areas of the substrate–scale interface where Nb_5_Si_3_ was present. A zone about 50 μm deep below the oxide scale consisted of Nb_ss_, Nb_5_Si_3_, A15–Nb_3_X and the π phase. This zone, together with the Sn-rich A15–Nb_3_X layer, comprised the subscale or diffusion zone (DZ). 

The oxide scale consisted of Nb and Ti-rich regions, Si-rich regions and Ti-rich regions (Table 5). The oxide scale was essentially free of Al, Fe and Sn. However, there were small areas in the scale that were rich in Sn. In the Si-rich regions, the Si/Ti and Nb/Ti ratios were about 1.4 and 1.1, respectively. In the Nb and Ti-rich regions and the Ti-rich regions, the Nb/Ti ratio was about 2.4 and 0.2, respectively. In the DZ, Nb_ss_ was severely oxidised (≤50 at.% oxygen) and exhibited “pitting” (see 12 and 16 in Figure 6b). Cracks parallel to the oxide scale surface formed in Nb_5_Si_3_ grains in areas up to about 10 μm below the scale. The Nb_5_Si_3_ silicide was the least oxidised among all the compounds, but no oxygen-free Nb_5_Si_3_ grains were observed. There was a small volume fraction of the π phase, which was also contaminated with oxygen, particularly near the scale (≤30 at.% oxygen). No “pitting” was observed in the A15–Nb_3_X grains, even though the latter phase was also severely oxidised near the scale (≤36 at.% oxygen). In Sn-rich A15–Nb_3_X, the oxygen concentration was about 32 at.%.

**Figure 7:**
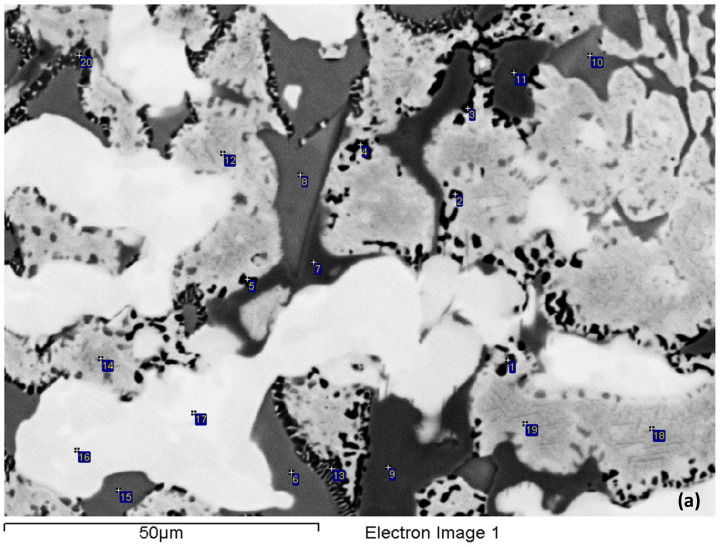

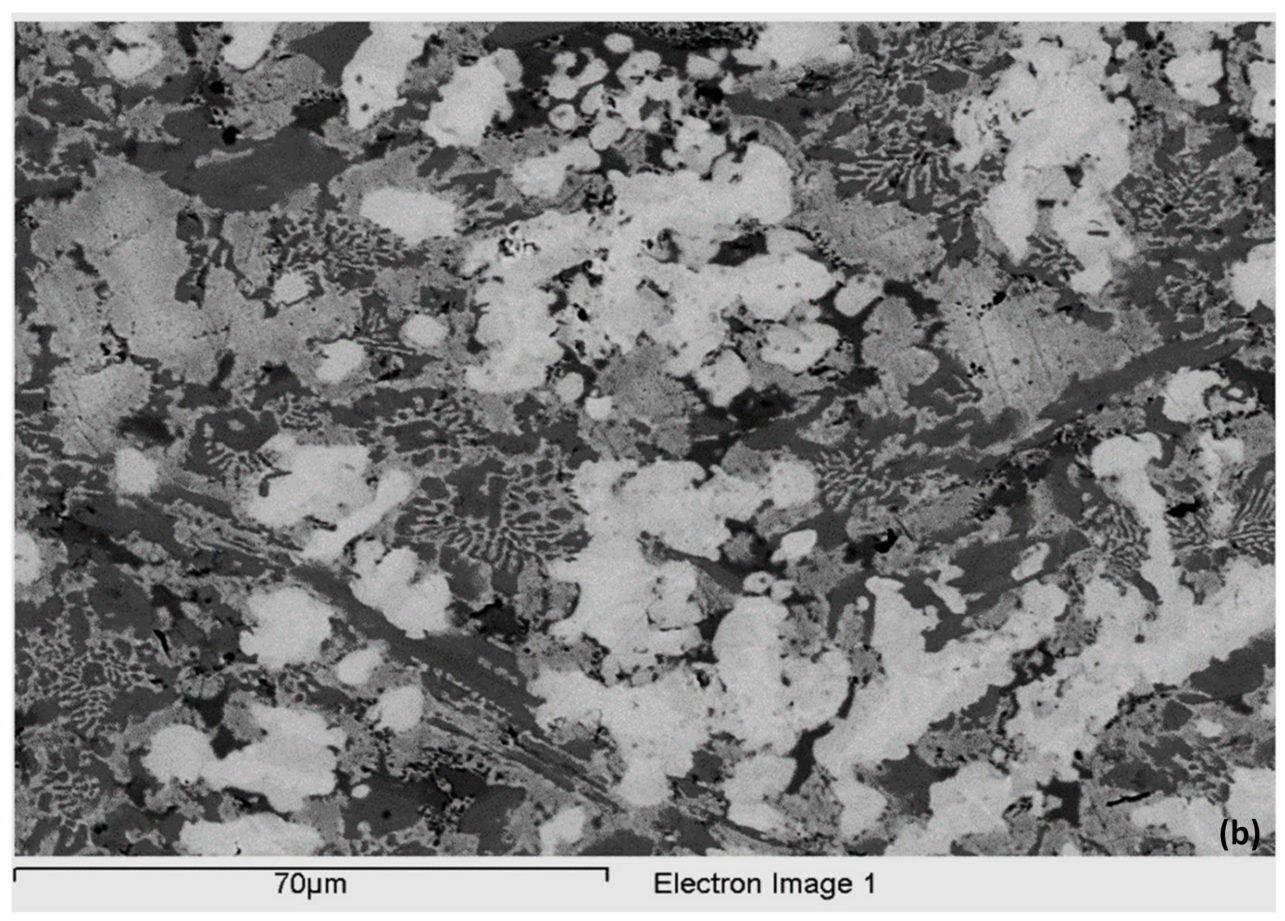
The microstructure of the bulk of NV2 after oxidation at 800 °C for 100 h. In (**a**), 1, 2, 3, 4 and 5 = Ti oxide; 8 and 10 = Nb_5_Si_3_; 14, 18 and 19 = Nb_ss_ and 7, 9 and 11 = the π phase. In (**b**), the bright phase is A15–Nb_3_X, the grey phase is Nb_ss_, the darker grey phase is Nb_5_Si_3_ and the very dark phase is the π phase. The contrast has been enhanced in (**b**) to show the Nb_ss_ + Nb_5_Si_3_ eutectic.

The microstructure from about 50 to about 300 μm below the oxide scale was also studied using EPMA (Figure 7 and Table 5) and consisted of the Nb_ss_, Nb_5_Si_3_ and π phases and a Ti-rich oxide, which was also observed in NV5–AC before and after oxidation. The Ti oxide was not observed in NV2–AC but formed after the isothermal oxidation and was unevenly distributed in the microstructure. Titanium oxide particles formed mainly around the π phase and at its interface with Nb_ss_, from which sometimes a lamellar microstructure of solid solution and Nb_5_Si_3_ was formed (Figure 7a). The aforementioned phases were contaminated with oxygen (Table 5), as was the case in the subscale. However, the oxygen content was reduced towards the bulk of the sample, as was the case in the oxidised NV1 [32] and NV5 (see below). In Nb_ss_, in which the oxygen concentration was as high as 15 at.%, no “pitting” was observed. Nb_5_Si_3_ was not cracked, and its average oxygen content was reduced slightly compared with the subscale. The lamellar microstructure of Nb_ss_ and Nb_5_Si_3_ seen in NV2-AC was still present (see Figure 7b). 

**Figure 8:**
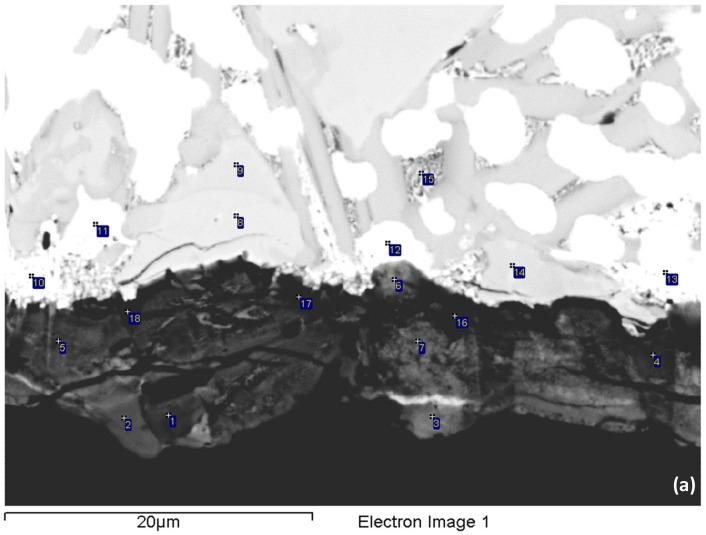

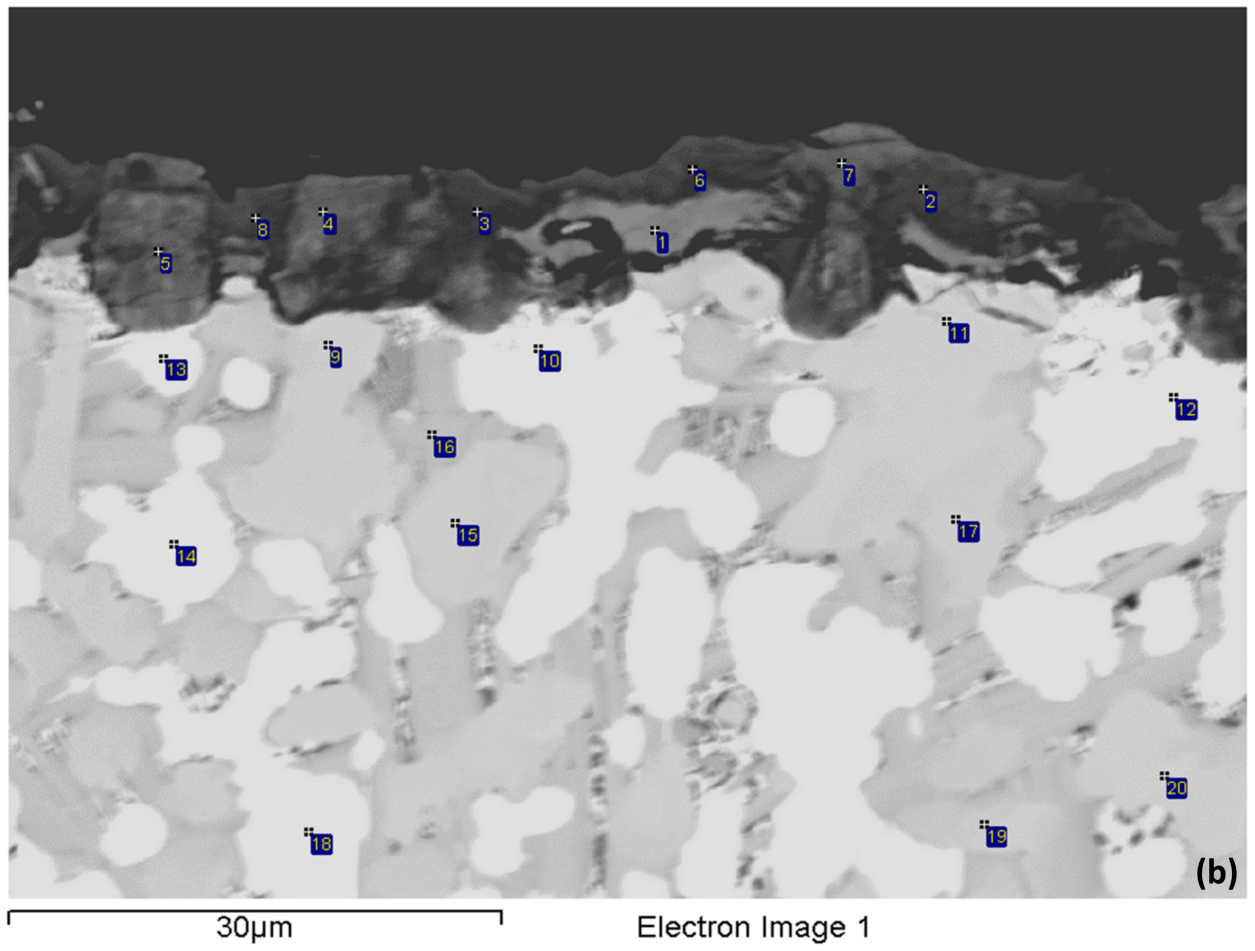
Oxide scale and subscale of NV5 after isothermal oxidation at 800 °C for 100 h. In (**a**), 1 and 4 = Si-rich oxide; 2 = mixed oxide, 16 and 17 = Ti-rich oxide; 3 and 7 = Ti and Nb-rich oxide; 8 and 9 = Nb_5_Si_3_; 10, 11, 12 and 13 = A15–Nb_3_X and 15 = Nb_ss_. In (**b**), 1 = Nb-rich oxide; 2, 3, 6 and 8 = Si-rich oxide; 4, 5 and 7 = Ti and Nb-rich oxide; 12, 13, 14 and 18 = A15–Nb_3_X and 9, 11, 15, 17, 19 and 20 = Nb_5_Si_3_.

The oxide scale of alloy NV5 was compact and relatively thin. Cracks parallel to the scale–substrate interface were observed, but the cracking of the scale was less severe compared with NV2. Cracks in Nb_5_Si_3_ were observed at the interface of the scale with the substrate, and the adherence of the oxide scale to the substrate was satisfactory. The oxide scale consisted (i) of Si-rich regions, where probably the Nb_5_Si_3_ or the Nb_3_Si silicides pre-existed, (ii) of Ti-rich regions, (iii) of Nb-rich regions and (iv) of Ti and Nb-rich regions with very low Si concentrations (<1 at.%), where probably Nb_ss_ pre-existed (Figure 8 and Table 6). There were also a few areas in the oxide scale that exhibited an intermediate contrast compared with the Si-rich and the Si-poor regions. In these areas, designated as mixed oxide (Figure 8 and Table 6), the Si concentration varied from 4 to 7 at.%, and the Si/Ti ratio was about 1. In the Si-rich regions, the ratios Si/Ti and Nb/Ti were about 2 and 2.6, respectively. In the Ti-rich regions, the Nb/Ti ratio was about 0.1; in the Nb-rich regions, about 5 and, in the Nb and Ti-rich regions, about 1.5. Overall, the Sn concentration in the scale was negligible, but there were a few very bright contrast thin layers in the oxide scale with high Sn contents (Figure 8), similar to alloy NV2. The concentrations of Fe and Cr in the oxides were also low. 

Just beneath and in contact with the oxide scale, there was a discontinuous zone that consisted of Sn-rich A15–Nb_3_X (about 21 at.% Sn), Nb_5_Si_3_, Nb_ss_ and the “prior” A15–Nb_3_X, meaning the compound that was formed in NV5–AC [22] and, less often, the FeNb_4_Si phase (Figure 8). The Sn-rich A15–Nb_3_X was either observed as discrete particles or was formed on top of the “prior” A15–Nb_3_X. All the phases were contaminated with oxygen, Nb_ss_ most severely (≤32 at.% oxygen). The Nb_5_Si_3_ silicide prevailed in the microstructure and exhibited little cracking in the near oxide scale–substrate region. 

**Figure 9:**
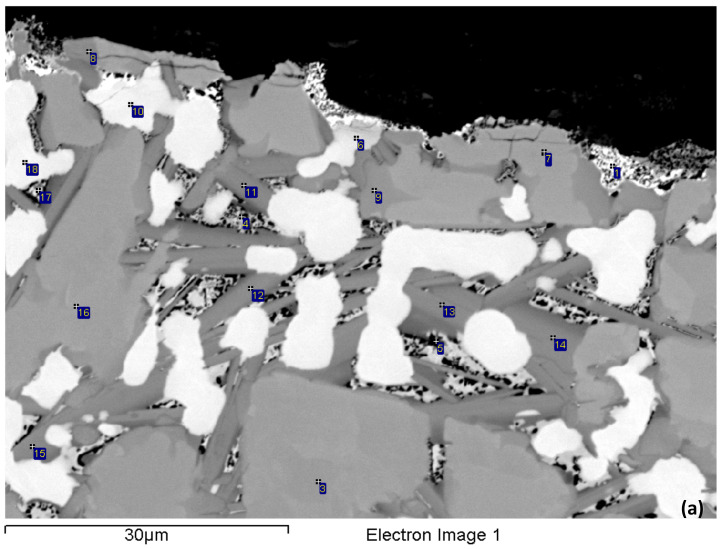

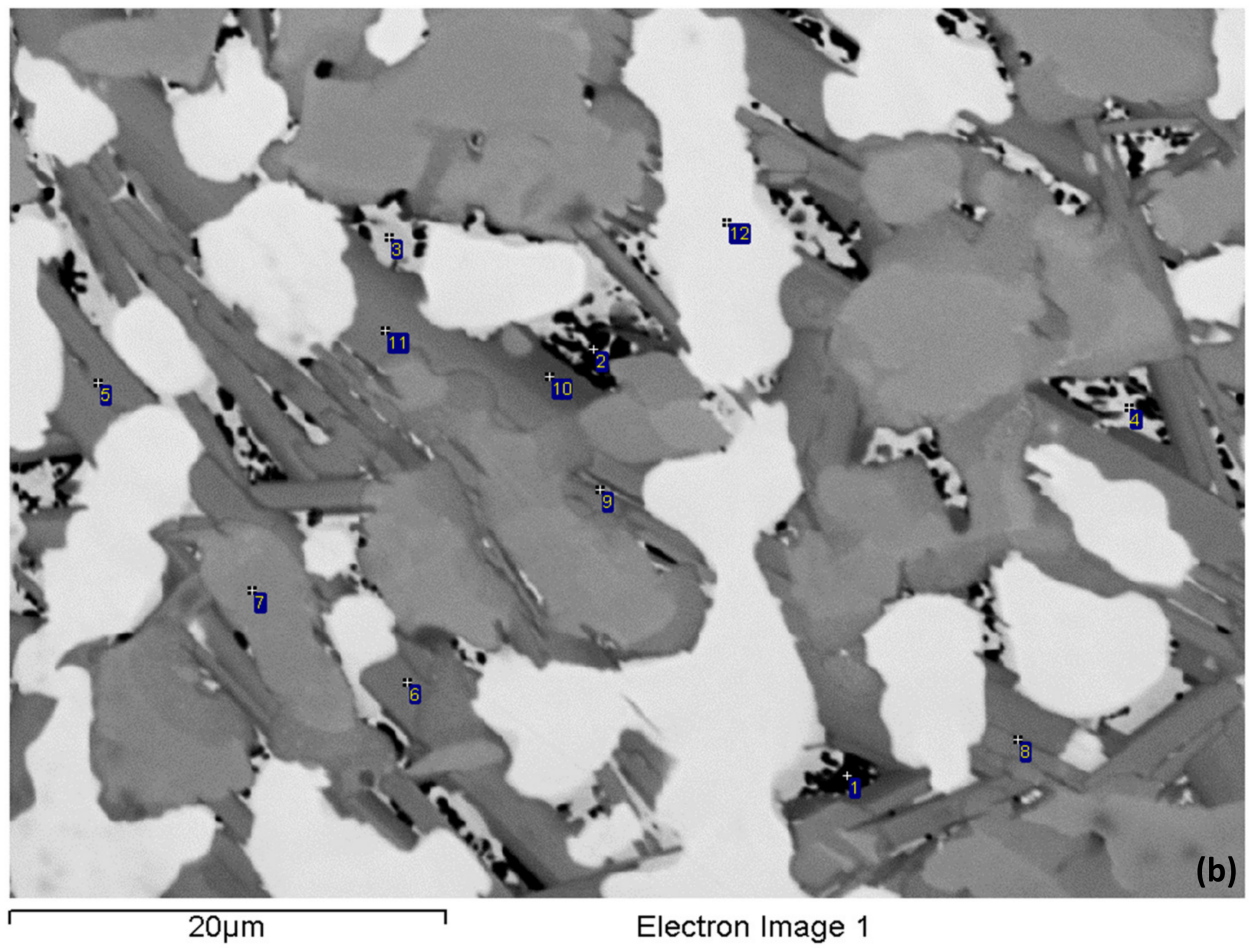
The microstructure of the bulk of NV5 after oxidation at 800 °C for 100 h. In (**a**), 1 = Sn-rich A15–Nb_3_X with oxide; 2, 3, 7, 8, 9 and 16 = Nb_5_Si_3_; 4 and 5 = Nb_ss_ with Ti oxide; 10 and 18 = A15–Nb_3_X and 11, 12, 13, 14 and 15 = FeNb_4_Si. In (**b**), 1 and 2 = Ti oxide; 3 = Nb_ss_; 5, 6, 8 and 11 = FeNb_4_Si; 7 = Nb_5_Si_3_; 10 = Fe_7_Nb_6_ and 12 = A15–Nb_3_X.

Towards the bulk of the alloy, the contamination of the microstructure became less severe (Table 6), and the microstructure consisted of the same phases as in the bulk of NV5–AC [22] (see Figure 9). The vol.% of the Fe_7_Nb_6_ phase and the 3-1 silicides was very low. There was an increase of the vol.% of Ti oxide that was found in the vicinity or in the Nb_ss_ but not near the FeNb_4_Si and Fe_7_Nb_6_ intermetallics. The vol.% of the Ti oxide near the oxide scale–substrate region was not as high as in the bulk of the alloy, where Nb_ss_ was present at a relatively higher vol.%.

## 4. Discussion

**Figure 10:**
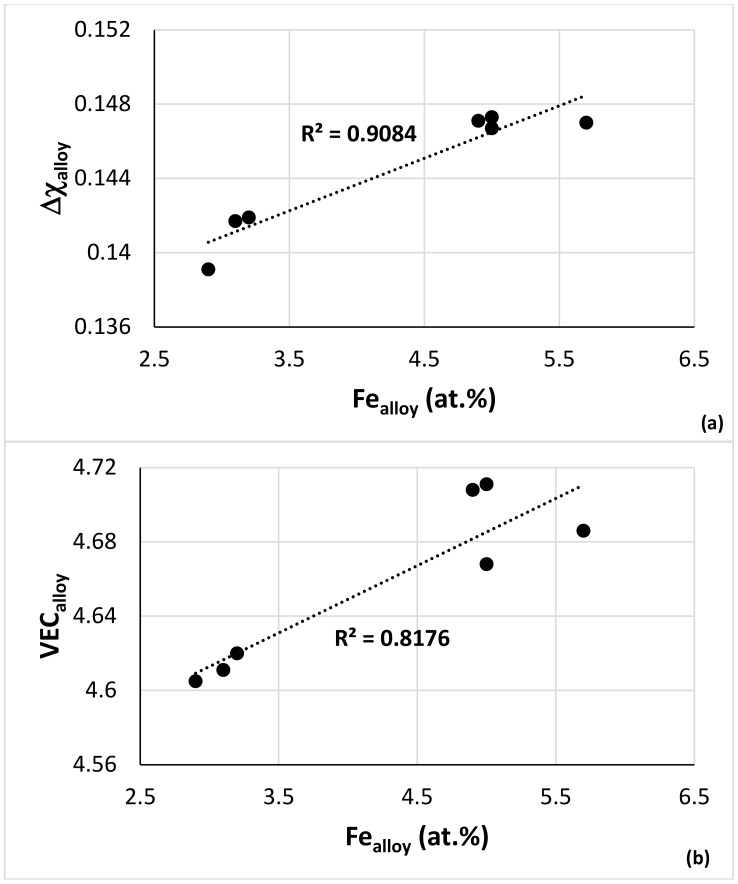
Relationships of Fe concentrations with the parameters (**a**) Δχ and (**b**) VEC in the alloys NV2, NV5 and NV8 (alloying elements Al, Cr, Fe, Hf, Nb, Si, Sn and Ti). R^2^ values for linear fit of data.

Alloy NV2 was designed using NICE [9] (see introduction). In NICE, there are relationships between the alloy parameters and the concentrations of the elements in RM(Nb)ICs and RM(Nb)ICs/RCCAs. The data for NV2–AC and NV2–HT, together with the data for alloys NV5 and NV8, were used to produce such relationships for the Fe concentration in Fe and Sn-containing alloys, examples of which are shown in Figure 10. 

Alloy NV2 was richer in Ti and poorer in Si than alloy NV5 (the actual composition of NV5–AC was 41.3Nb–25.3Ti–18.6Si–4.9Cr–5Fe–4.9Sn) [22]. Similar to alloy NV5, alloy NV2 was heat-treated at 1200 °C, which is different compared with many Nb–24Ti–18Si-based RM(Nb)ICs that do not suffer from liquation at higher temperatures. Furthermore, the Ti and Hf concentrations in NV2 gave Nb/(Ti + Hf) = 1.35 for the nominal composition (1.32 for the calculated composition), which would suggest poor creep properties at the creep goal conditions [3,5,6,9], given that this ratio should be above 2 for a low secondary creep rate [8]. In other words, alloy NV2 should be “evaluated” only on whether it meets the oxidation goal that guided its design using NICE [9]. 

Below, alloy NV2 is compared with alloy NV5 and other RM(Nb)ICs where appropriate to understand the effect of the synergy of Fe with Al, Cr, Hf and Sn on the microstructure and properties. Guided by NICE, data for the Fe-containing alloys NV3, NV5, NV2 and NV8 are used to show relationships between solutes in different phases formed in NV2 that are used to develop the Fe database for metallic UHTMs in NICE with relationships like those shown in Figure 10.

### 4.1. Microstructure

#### 4.1.1. Macrosegregation

Tin at 5 at.% concentration and in synergy with Al and Cr significantly increased the macrosegregation of Si (MACSi) in Nb–24Ti–18Si-based RM(Nb)ICs [28]. MACSi was reduced slightly with the addition of Hf [30]. When Fe was added in Nb–24Ti–18Si-based RM(Nb)ICs, MACSi was increased (compare to alloys KZ4 and NV3 in Table 7), and MACSi increased more when Sn was added in alloy NV5 (compare to alloys NV3 and NV5 in Table 7). In other words, in Nb–24Ti–18Si-based RM(Nb)ICs, the individual addition of Fe or Sn increased MACSi, whereas the simultaneous addition of Fe and Sn reduced MACSi. Furthermore, the synergy of Fe and Sn with Al, Cr and Hf in NV2–AC also reduced MACSi, compared with NV5–AC, which is consistent with the “direction” of change of MACSi, as discussed in [34] (also see [28]). To put this another way, with the simultaneous addition of Al, Cr, Fe, Hf, Sn and Ti, of which Al, Cr, Hf, Sn and Ti are key solutes for oxidation resistance [6,9] (the role of Fe is “disputed”; see Introduction), it is possible to cast RM(Nb)ICs with low MACSi but at the expense of the liquation at temperatures above 1200 °C. 

#### 4.1.2. Nb Solid Solution 

The partitioning of Ti to Nb_ss_ and the formation of Ti-rich Nb_ss_, and the stability of Nb_ss_ in RM(Nb)ICs, are important (i) for the contamination with oxygen of near the surface areas and the bulk of alloys, (ii) for the oxidation of the alloys at pest and higher temperatures [27,28,32] and (iii) for the creep of the alloys [9,13] and are known to be affected by the individual solute additions that were used in the alloy NV2 and, in particular, (a) by the elements that are present simultaneously in an alloy and (b) by the concentration of Sn in the alloy. A summary of the relevant data for Nb–24Ti–18Si-based RM(Nb)ICs is given in Table 8. Regarding Ti-rich Nb_ss_, in Fe-free RM(Nb)ICs, where Ti and Si were in synergy with/without Al and/or Cr and with/without 2 at.% Sn—namely, alloys KZ3, KZ4, KZ5, KZ7, ZX3, ZX5 and ZX7 (see Table 8 for the nominal compositions)—Nb_ss_ and Ti-rich Nb_ss_ formed in the cast microstructures and Nb_ss_ were stable after heat treatment. Titanium-rich Nb_ss_ did not form in the alloys where Ti and Si were in synergy with/without Al and/or Cr and with 5 at.% Sn addition—namely, alloys NV6, ZX4, ZX6 and ZX8 or when Hf and 5 at.% Sn were simultaneously present with Al (alloys EZ5 and EZ8). Additionally, note that Ti-rich Nb_ss_ did not form in the heat-treated condition of the alloys in Table 8.

Earlier works reported that Fe in RM(Nb)ICs had an effect on the formation of Ti-rich Nb_ss_ only when it was in synergy with Cr and 5 at.% Sn (alloy NV5). Nb_ss_ was not stable when Al and 5 at.% Sn were in synergy (alloy ZX6), when Hf and 5 at.% Sn were simultaneously present with Al (alloys EZ5 and EZ8) and when Fe was in synergy with 5 at.% Sn with/without Cr (alloys NV5 and NV8). In other words, 5 at.% Sn and specific solutes from the group of the elements Al, Cr, Fe and Hf can support or suppress (1) the formation of Ti-rich Nb_ss_ in cast RM(Nb)ICs and (2) the stability of Nb_ss_ in the alloys. The data for alloy NV2 would suggest that, with 5 at.% Sn, the concentrations of Al, Fe, Hf, Si and Ti in RM(Nb)ICs could be key for the formation of Ti-rich Nb_ss_ and the stability of Nb_ss_. 

**Figure 11:**
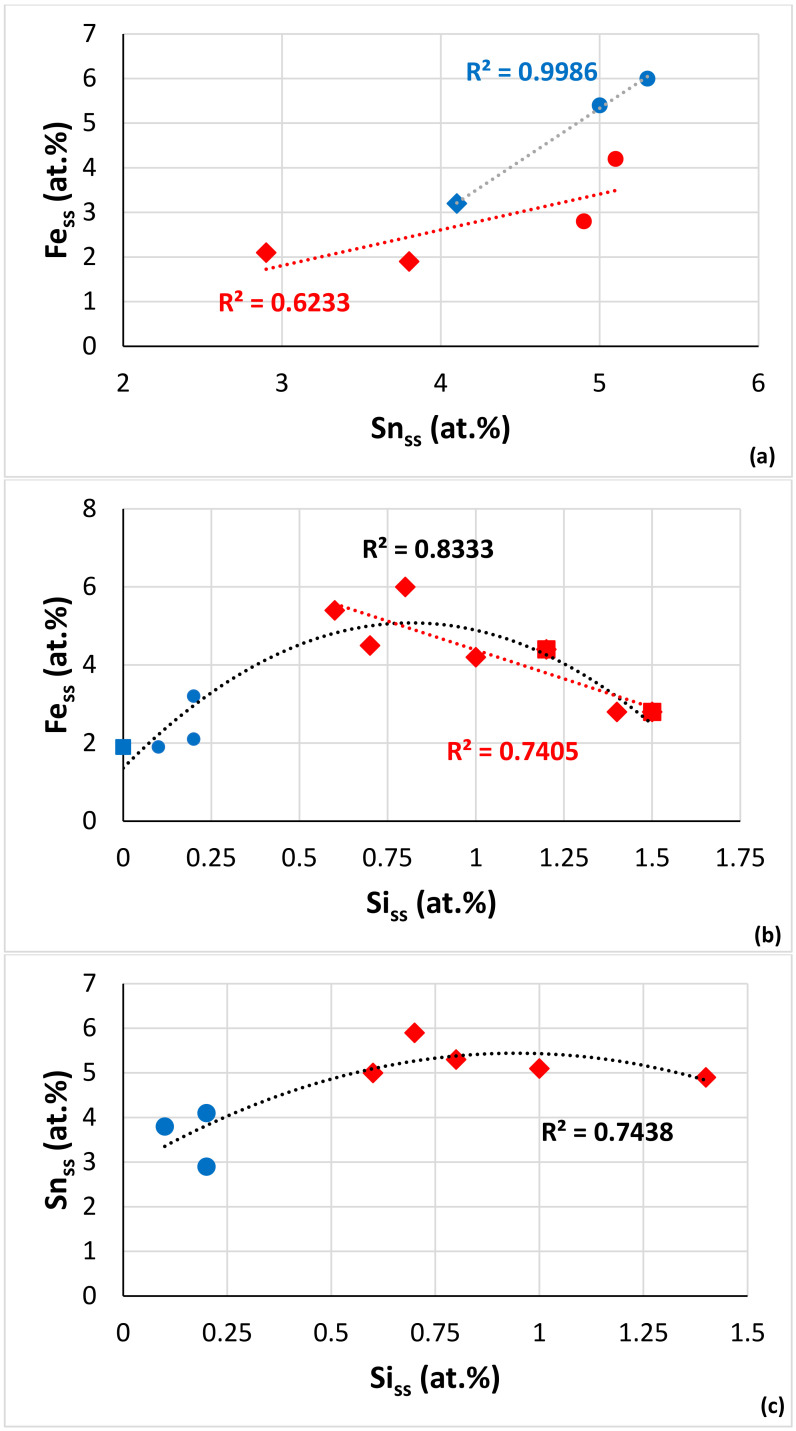

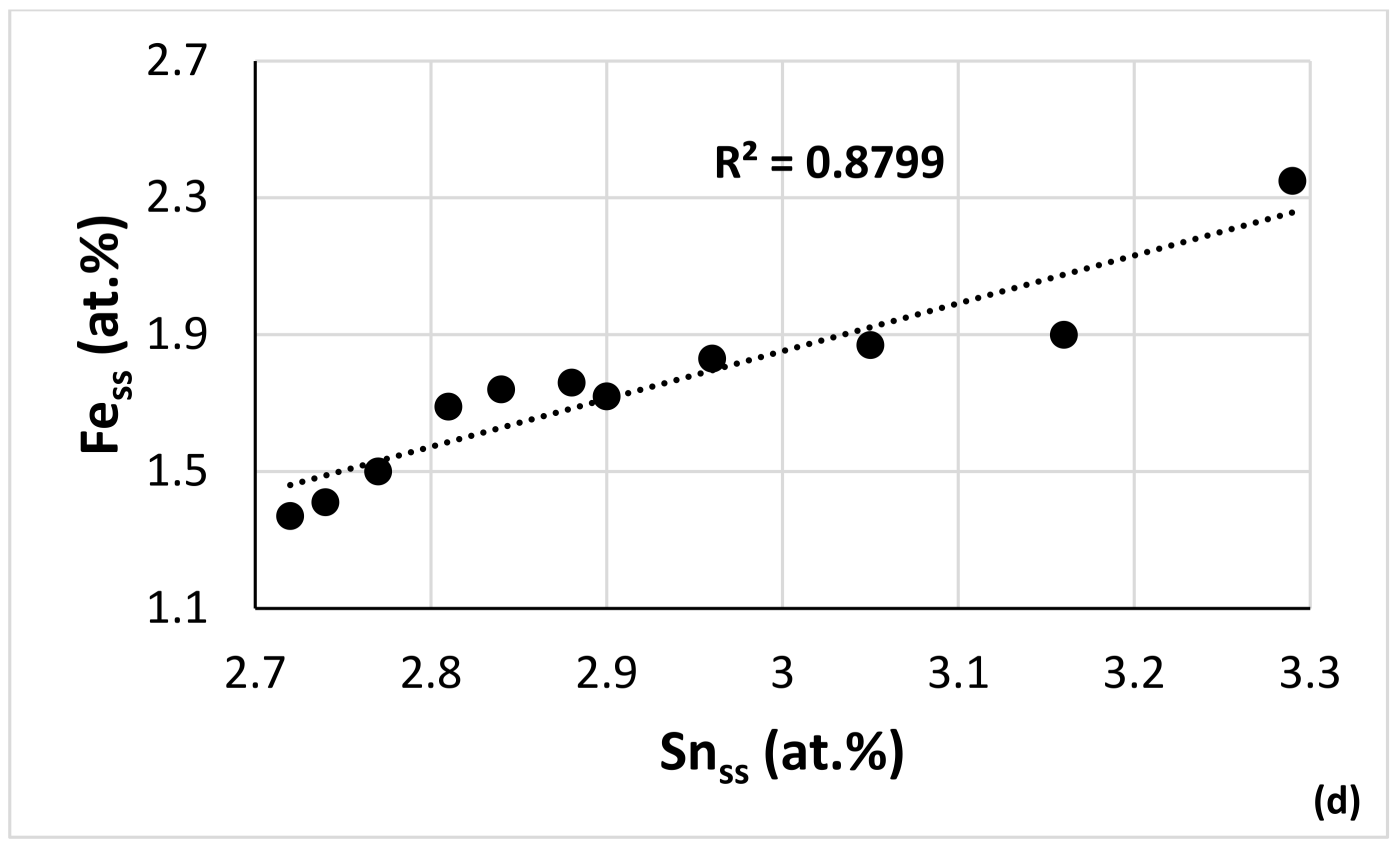
Data for Nb_ss_ in Fe-containing RM(Nb)ICs. (**a**) Fe versus Sn, (**b**) Fe versus Si and (**c**) Sn versus Si content in Nb_ss_. For the data used in (**a**–**c**), see the text. In (**a**), alloy NV2 (red data) and alloy NV8 (blue data) are diamonds for the average chemical composition of the solid solution in the heat-treated microstructures. In (**b**), the data are for alloys NV3, NV5, NV8 [22,31] and NV2; for all data, R^2^ = 0.8333, the red data (R^2^ = 0.7405) are for Nb_ss_ in the as-cast alloys, the blue data are for Nb_ss_ in the heat-treated alloys and the squares are for the Sn-free alloy NV3. In (**c**), the diamonds are for the cast alloys and circles for the heat-treated alloys. In (**d**), the data are only for the grains of Nb_ss_ with no Si in NV2–HT1.

Owing to the very limited chemical analysis data for Fe-containing RM(Nb)ICs with a Sn addition, we used the data for the solid solution in NV2–AC (Table 3) and the data for the average chemical composition of the solid solutions in NV2–HT1 and NV2–HT2 (see the end of Section 3.2) with the data for the solid solution in the alloys NV3, NV5 [22] and NV8 [31] to find out if there are trends regarding the concentrations of Fe, Si and Sn in the solid solution. Figure 11a would suggest that the Sn solubility in the solid solution increases with the increasing Fe content. Additionally, the data showed (i) that, in the cast microstructures, the solubility of Si in the solid solution decreased as the Fe content increased (Figure 11b), (ii) that the Si concentration in the solid solution in the heat-treated microstructures would be very low (Figure 11b,c) and (iii) that the concentrations of Fe and Sn would be low in the solid solution after heat treatment (Figure 11a–c). The parabolas in Figure 11b,c would suggest “maximum” solubilities of Fe and Sn in the solid solution in cast RM(Nb)ICs, respectively, 5 and 5.4 at.%. 

##### Nb Solid Solution with No Si

The trend shown in Figure 11a was supported by the data for Nb_ss_ with no Si—for example, see Figure 11d, where the data are only for the grains of Nb_ss_ with no Si in NV2–HT1. Previously, our research group reported that, in Fe-free RM(Nb)ICs with RM additions, Nb_ss_ can be Si-free (according to the chemical analysis of Si using EPMA) [12]. In other words, “normal” Nb_ss_ and/or Nb_ss_ with no Si can form in the cast microstructure, and Nb_ss_ with no Si can be stable after the heat treatment. This was observed (a) in Ti-free RM(Nb)ICs without Ge or Sn additions, where RM = Mo, Ta and W [35,36,37], and (b) in Ti-containing RM(Nb)ICs with low or high Ti concentrations and with simultaneous Ge and Sn additions, where RM = Mo and W [38] (also see Table 1 in [12]). Nb_ss_ with no Si in (b) was rich in RM and poor in Ti content, owing to the partitioning behaviour of Mo, Ti and W in these RM(Nb)ICs [5,38]. Furthermore, for the alloys in (a) and (b), parameter δ of the solid solution separated Nb_ss_ with no Si from Ti-rich Nb_ss_; the former had δ less than about 5, and the latter, which was observed only in the cast microstructures, had δ higher than about 5 [5,6,12]. 

The data for the Fe-containing but RM-free RM(Nb)ICs alloys NV2, NV3, NV5 and NV8 [22,31] showed (i) that only Nb_ss_ with no Si was stable in NV3–HT (Nb_ss_ = 60.5Nb–30.9Ti–0Si–6.7Cr–1.9Fe [22]), (ii) that “normal” Nb_ss_ and Nb_ss_ with no Si were stable in NV2–HT1 and NV2–HT2 (Table 3), (iii) that “normal” Nb_ss_ and Nb_ss_ with no Si formed in NV5–AC [22] and (iv) that only “normal” Nb_ss_ formed in NV8 [31]. Nb_ss_ with no Si in (i) and (ii) was rich in Ti compared with Nb_ss_ with no Si in Fe-free RM(Nb)ICs (see (a) and (b) in the previous paragraph) and also had δ less than 5 (3.942, 4.135 and 4.688, respectively, for NV3–HT, NV2–HT1 and NV2–HT2), whereas, in NV2–HT, “normal” Nb_ss_, which was richer in Ti than (Ti-rich) Nb_ss_ with no Si (Table 3), had δ ≥ 5 (4.993 and 5.086, respectively, for NV2–HT1 and NV2–HT2). Furthermore, the Ti-rich Nb_ss_ in NV2–AC had δ = 5.61, in agreement with [5,6,12]. In other words, the limited data for Fe-containing RM(Nb)ICs would suggest that these alloys comply with the “δ parameter rule” in NICE (see Figure 15 in [9]) regarding the Si content in the bcc solid solution. 

Considering the chemical compositions of alloys NV2, NV3, NV5 [22] and NV8 [31], the stability of Nb_ss_ with no Si in Fe-containing RM(Nb)ICs was attributed to the synergy of Fe with Cr and Ti (alloy NV3), whereas, when Cr was substituted with Sn in NV8, the synergy of Fe with Sn and Ti did not affect the partitioning of Si in the solid solution (no Si-free Nb_ss_ was formed in NV8), but when Sn was added in NV5 and Al and Hf were added in NV2, the effectiveness of the synergy of Fe with Cr and Ti was reduced (“normal” Nb_ss_ and Nb_ss_ with no Si were observed in NV2 and NV5; see previous two paragraphs). Furthermore, the data for the solid solutions in NV2–HT1 and NV2–HT2 provided further experimental evidence in support of the conclusion in [39] that the concentrations of Al and Cr in the solid solution increase with its Ti concentration.

Silicon-free Nb_ss_ in Nb–silicide-based alloys (i.e., RM(Nb)ICs, RM(Nb)ICs/RCCAs and RM(Nb)ICs/RHEAs) is desirable, owing to the anticipated yield stress and toughness of this solid solution [5]. Chromium and Ti are known to have a strong effect on the toughness of Nb–Ti–Cr solid solution alloys, which was reduced with the addition of Al [40,41]. In Nb–Ti–Al–Cr bcc solid solution alloys, the Nb/Ti ratio and the (Al + Cr) sum are important for ductility and the (Al + Cr) sum for the room temperature yield strength [5]. For low Nb/Ti ratios, “more (A l+ Cr) is permissible” before brittleness is observed. For example, if Nb/Ti = 1.3, then (Al + Cr) should be below 20 at.% to get ductility [42]. The Nb/Ti ratio was 1.5 and 1.2 for Nb_ss_ with no Si and 1.25 and 1.04 for “normal” Nb_ss_, and the (Al + Cr) sum was 5.9 and 7.7 at.% for Nb_ss_ with no Si and 7.6 and 9 at.% for “normal” Nb_ss_, respectively, for NV2–HT1 and NV2–HT2, which points toward a ductile solid solution in NV2.

To recap, it is possible to have stable Nb_ss_ with no Si in RM(Nb)ICs (1) with RM additions (RM = Mo and W when Ti is present simultaneously with Ge and Sn in the alloy; in which case, the solid solution is poor in Ti or RM = Mo, Ta or W in the absence of Ti, Ge or Sn in the alloy) or (2) with Fe and Cr additions (plus the addition of Sn with Al and Hf) in Ti-containing alloys; in which case, the solid solution would be rich in Ti. Route (2) makes it possible to engineer oxidation-resistant solid solutions, owing to their high Ti content [43,44]; with ductility, owing to their Nb/Ti ratio and Al + Cr sum; and with additions of Al, Cr, Fe, Hf and Sn in RM(Nb)ICs. 

#### 4.1.3. Nb_5_Si_3_ Silicide 

In RM(Nb)ICs, the partitioning of solutes to Nb_5_Si_3_ is important, because it affects (a) the properties of the silicide [7,9,13,30,32,45,46,47], (b) the properties of the Nb_ss_/Nb_5_Si_3_ interface [32], (c) the silicide crystal structure (meaning tetragonal α or β Nb_5_Si_3_ or hexagonal γNb_5_Si_3_ [11,33]), (d) the contamination of Nb_5_Si_3_ with oxygen [27,28,32] and € the oxidation and creep of the alloys [5,7,9,13]. In Fe and Sn-free RM(Nb)ICs, Ti-rich Nb_5_Si_3_ was formed in the cast alloys together with Nb_5_Si_3_, and in most alloys, both were stable after heat treatment(s) (e.g., [15,16]). When Hf was added to RM(Nb)ICs with 5 at.% Sn, the Ti-rich Nb_5_Si_3_ was also rich in Hf [30]. In Fe-free and Sn-containing RM(Nb)ICs, the effect of Sn on the partitioning of Ti depended on the Sn concentration in the alloy (e.g., alloys ZX5, ZX7 [27] and ZX4 [28], where Ti-rich Nb_5_Si_3_ was not stable after the heat treatment; see Table 8 for the nominal alloy composition) and other solute additions (e.g., the alloy ZX3 [27] and the alloys ZX6 and ZX8 [28], where Sn was in synergy with Cr (ZX3) or Al (ZX6) or Al and Cr (ZX8)). In Fe and 5 at.% Sn-containing alloys NV5 and NV8, Ti-rich Nb_5_Si_3_ was not observed after the heat treatment [22,31]. In other words, in RM(Nb)ICs with the solute additions used in NV2, one would expect a partitioning of Ti and Hf to Nb_5_Si_3_. Indeed, this was the case in NV2-AC, where the partitioning of the said elements resulted in tetragonal Nb_5_Si_3_ (the Nb/(Ti + Hf) ratio was equal to 1.13 and 1.75, respectively, for Nb_5_Si_3_ and Ti-poor Nb_5_Si_3_) and hexagonal Ti and Hf-rich Nb_5_Si_3_ with Nb/(Ti + Hf) = 0.83 (Table 3), whereas, in NV2–HT1 and NV2–HT2, hexagonal Nb_5_Si_3_ was stable, with Nb/(Ti + Hf) about 0.7 and 0.8, respectively.

**Figure 12:**
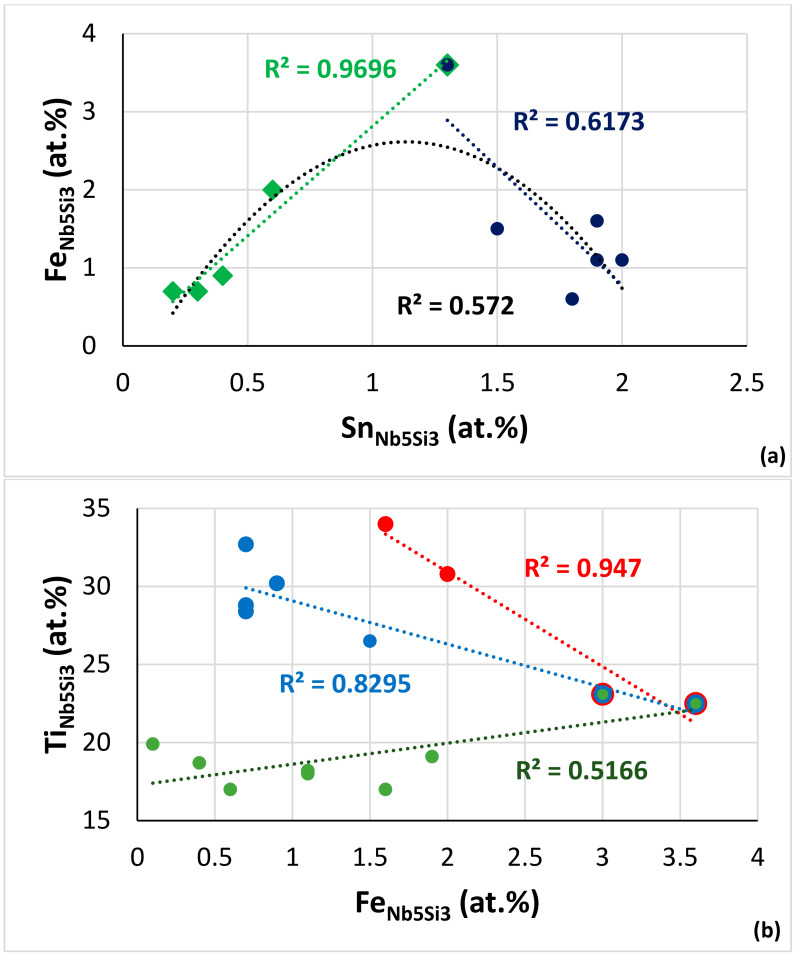

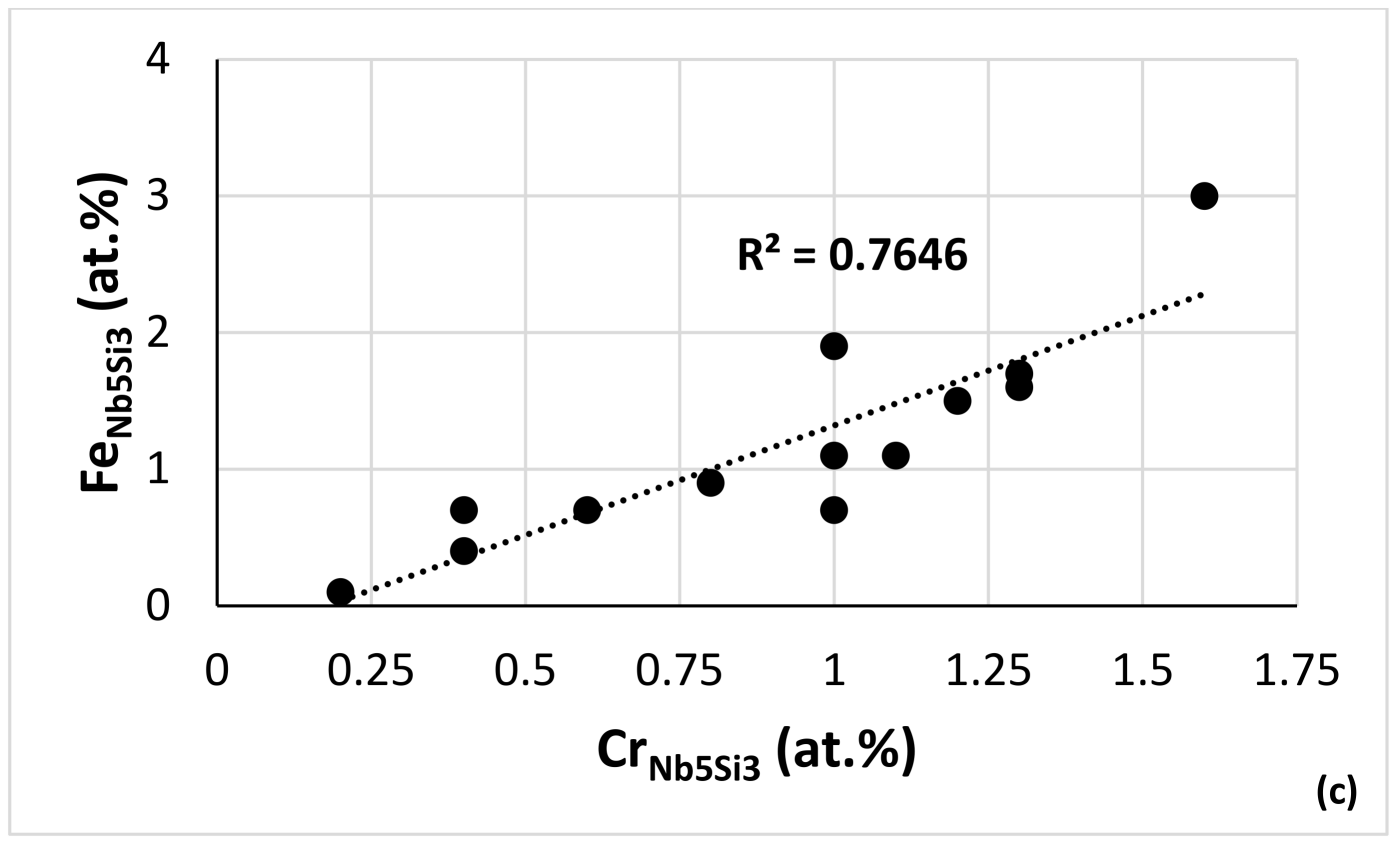
(**a**) Fe versus Sn, (**b**) Ti versus Fe and (**c**) Fe versus Cr in Nb_5_Si_3_. Data for alloys NV2, NV5 and NV8 in (**a**); for NV2, NV3, NV5 and NV8 in (**b**) and for NV2, NV3 and NV5 in (**c**). In (**a**), R^2^ = 0.572 for the parabolic fit of the data. Intercept of the lines in (**b**) corresponds to Fe = 3.3 at.% and Sn = 1.2 at.%.

In NV2–AC, the average concentration of Hf in Nb_5_Si_3_ and Ti-poor Nb_5_Si_3_ was the same (2.8 at.%), but as the Ti concentration decreased, so did the concentrations of Al, Cr, Fe and Sn. The interdependence of the solubility of Ti and the solubilities of Al, Cr and Fe in Nb_5_Si_3_ was also supported by the EPMA data for the Hf-rich Nb_5_Si_3_. The comparison of Nb_5_Si_3_ and Hf-rich Nb_5_Si_3_ showed that, as the Hf solubility increased, the solubility of Sn decreased significantly, and also, there was a small decrease of the solubility of Fe. It is suggested that the solubilities of Al, Cr and Fe in Nb_5_Si_3_ in NV2 were controlled by Ti and the solubility of Sn by Hf. The Fe versus Sn concentration figure for Nb_5_Si_3_ in the alloys NV2, NV5 and NV8 suggests a maximum Fe concentration (Figure 12a). The parabolic fit of all the data has R^2^ lower than the R^2^ values of the linear fits of the two parts of the data. The maximum of the parabola corresponds to Fe = 2.6 at.% and Sn = 1.1 at.%, whereas the intercept of the lines gives Fe = 3.3 at.% and Sn = 1.2 at.%. The Ti versus Fe concentration figure for the same alloys plus alloy NV3 shows that the data converges to a maximum Fe concentration of 3.3 at.% (Figure 12b). The Fe concentrations in Nb_5_Si_3_ from this work are in very good agreement with the data for (Nb,Fe)_5_Si_3_ in [21]. The Cr content in Nb_5_Si_3_ increased with its Fe concentration (Figure 12c). For the maximum Fe content, the Cr concentration in the silicide is about 2.3 at.%.

#### 4.1.4. A15–Nb_3_X Compound 

**Figure 13:**
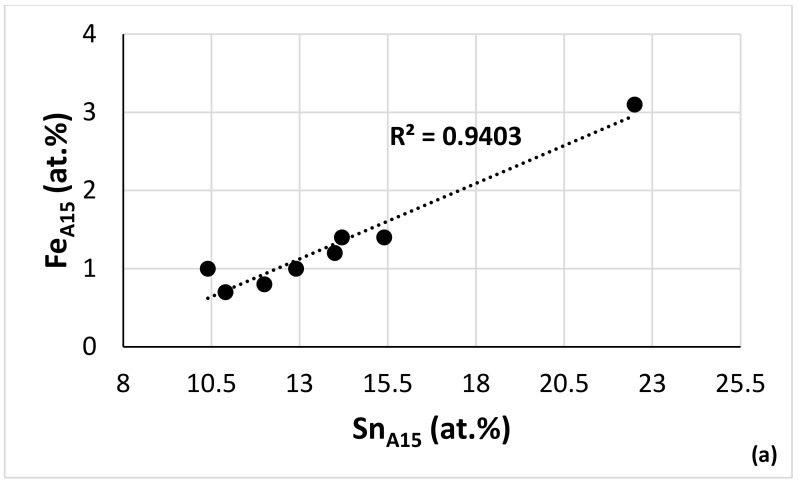

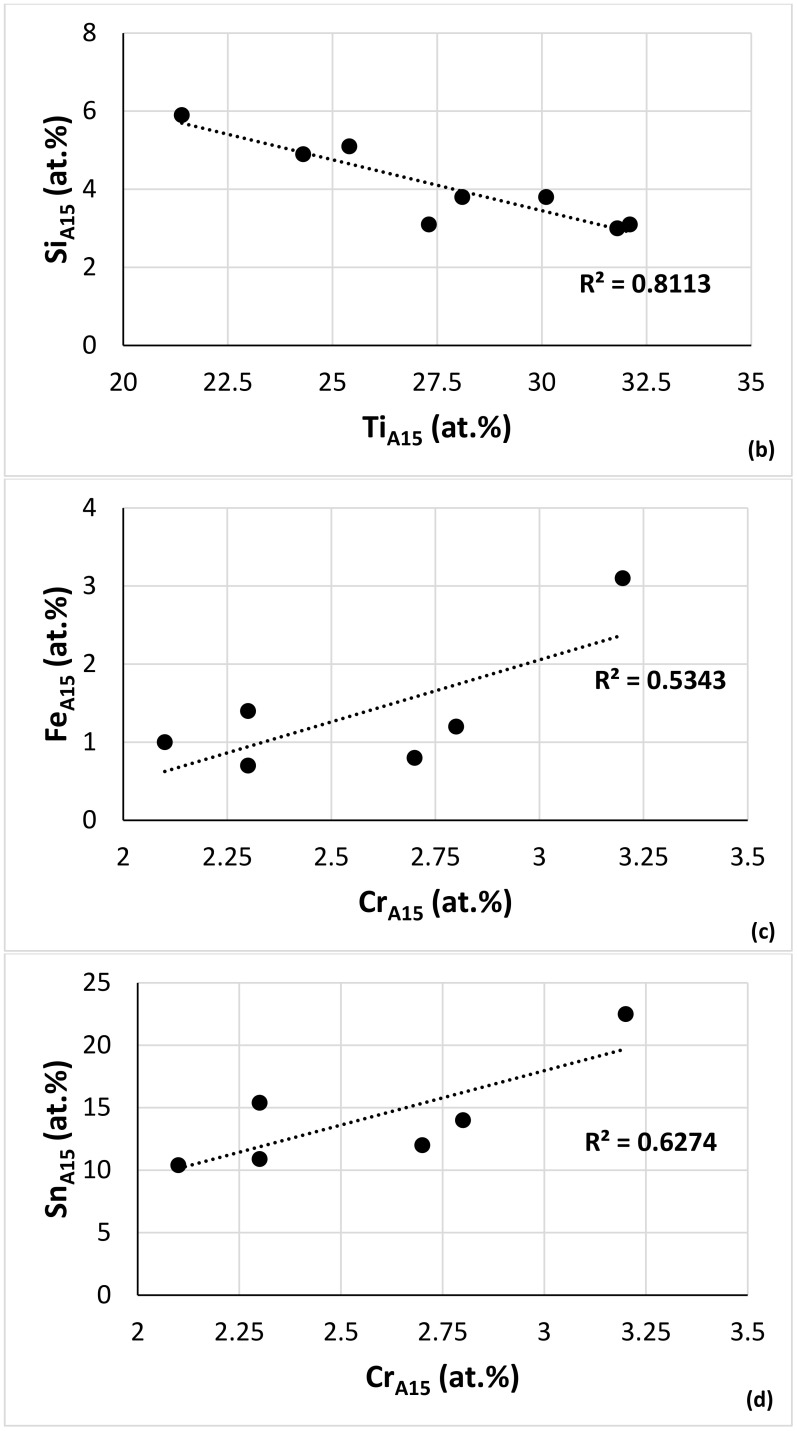
Data for solutes in A15–Nb_3_X in alloys NV2, NV5 [22] and NV8 [31]. (**a**) Fe versus Sn and (**b**) Si versus Ti. In (**c**–**e**), correlations of the Cr concentration with Fe in (**c**), with Sn in (**d**) and with Si in (**e**). The parabolic fit of the data in (**c**–**e**) gives R^2^ = 0.8878 in (**c**), R^2^ = 0.7874 in (**d**) and R^2^ = 0.915 in (e) with the minima of the parabolas corresponding to Cr concentrations 2.45, 2.33 and 2.44 at.% in (**c**–**e**), respectively, and Fe = 0.75 at.%, Sn = 11.5 at.% and Si = 2.9 at.% in (**c**–**e**), respectively.

A15–Nb_3_X was formed at a significant vol.% and had areas richer and poorer, respectively, in Ti and Sn, similar to alloys NV5, NV6 and NV8 [16,22,31]. The formation of A15–Nb_3_X in NV2–AC and the stability of this phase after heat treatment was expected in alloy NV2 according to NICE and previous research on RM(Nb)ICs with a 5 at.% Sn addition (see Table 1) and was confirmed by the experimental results of this research. The Fe concentration in A15–Nb_3_X increased with the increasing Sn content (Figure 13a) and with the Si content (figure not shown), whereas, with the increasing Ti concentration in A15–Nb_3_X, the Si content decreased (Figure 13b). With the increasing Cr concentration in A15–Nb_3_X, the concentrations of Fe, Sn and Si increased (Figure 13c–e). The data would suggest minimum concentrations of Cr, Fe, Si and Sn in A15–Nb_3_X, respectively, 2.4, 0.75, 2.9 and 11.5 at.% (see Figure 13 caption).

#### 4.1.5. 3-1 Silicides

The experimental results confirmed that both tP32 Nb_3_Si and metastable Nb_3_Si-m’, which, respectively, were stable when Fe was in synergy with 5 at.% Sn in alloy NV8 [31] or with 5 at.% Sn and 5 at.% Cr in alloy NV5 [22] and were stable in alloy NV2. This was attributed to the simultaneous addition of said elements with Al and Hf, which, in synergy, suppressed Nb_3_Si [47]. In Nb_3_Si, the solubility of Ti was similar to that in NV5–AC, but the Cr and Fe concentrations decreased dramatically. The sensitivity of the formation of Nb_3_Si and Nb_3_Si-m’ to solidification conditions was attributed, respectively, to the addition of Fe [16,31] and to the synergy of Al and Hf in NV2–AC. Nb_3_Si-m′ in NV2–AC was poorer in Ti, Cr and Sn than in NV5–AC [22]. In other words, this research (a) confirmed that Nb_3_Si cannot be suppressed in Fe-containing RM(Nb)ICs with the alloying additions that are essential to improve oxidation behaviour—namely, Al, Cr, Hf, Sn and Ti—and (b) showed that, in RM(Nb)IC with the said alloying additions, Nb_3_Si did not transform into Nb_ss_ and αNb_5_Si_3_. The latter would suggest that it is unlikely to engineer the microstructure of RM(Nb)ICs with a Fe addition using the eutectoid transformation Nb_3_Si → Nb_ss_ + αNb_5_Si_3_. Could a Nb_ss_ + Nb_5_Si_3_ eutectic be brought about in the cast microstructure?

#### 4.1.6. Eutectic 

The suppression of Nb_3_Si by Sn in the Fe-free RM(Nb)IC alloys NV9 (Nb–18Si–5Sn), NV6 (Nb–24Ti–18Si–5Sn) [16], EZ1 (Nb–18Si–5Hf–5Sn), EZ4 (Nb–18Si–5Al–5Hf–5Sn) [48] and EZ2 (Nb–24Ti–18Si –5Hf–5Sn), EZ5 (Nb–24Ti–18Si–5Al–5Hf–5Sn) [30] replaced Nb_ss_ + Nb_3_Si eutectic with Nb_ss_ + βNb_5_Si_3_ eutectic. The addition of Fe in NV8 (Nb–24Ti–18Si–5Sn–5Fe), which stabilised Nb_3_Si, suppressed the latter eutectic but did not favour the former one. When Fe was in synergy with Cr in the Sn-free alloy NV3 (Nb–24Ti–18Si–5Cr–5Fe) or with Cr and Sn in alloy NV5 (Nb–24Ti–18Si–5Cr–5Fe–5Sn), tP32 Nb_3_Si was formed in the cast microstructures of both alloys but not the Nb_ss_ + βNb_5_Si_3_ eutectic. This data suggested that, in Nb-24Ti-18Si-based RM(Nb)ICs with Fe and Sn additions, the Nb_ss_ + Nb_3_Si eutectic would not form even in the presence of Nb_3_Si in the cast microstructure. However, when the concentration of Ti was increased in the Fe and Sn-containing RM(Nb)IC NV4 (Nb–45Ti–15Si–5Fe–5Sn), the (Nb,Ti)_5_Si_3_ and (Ti,Nb)_5_Si_3_ and (Nb,Ti)_3_Si and (Ti,Nb)_3_Si silicides (meaning Nb-rich or Ti-rich 5-3 or 3-1 silicides; note that Nb_3_Si and Ti_3_Si have the same structure but not the Nb_5_Si_3_ and Ti_5_Si_3_ [11]) were formed, as well as a eutectic of bcc (Ti,Nb)_ss_ (meaning Ti-rich bcc solid solution with Ti/Nb > 1) with Nb_5_Si_3_ silicide. The latter research suggested that a high concentration of Ti in Fe and Sn-containing RM(Nb)IC would favour the eutectic of the bcc solid solution with 5-3 silicide, even in the presence of 3-1 silicide. The increase of the Ti concentration from 24 at.% to 30 at.% Ti in alloy NV2 confirmed this to be the case, as Nb_ss_ + Nb_5_Si_3_ eutectic was formed in NV2–AC. This eutectic was stable after 100 h at 800 °C (Figure 7b). Furthermore, the chemical composition of the eutectic corresponded to a complex concentrated (compositionally complex) eutectic [7] and its <Si> = Al + Si + Sn content was in agreement with [49].

#### 4.1.7. Cr Rich Intermetallic Phase 

In Fe-free but Sn-containing RM(Nb)ICs, the C14–NbCr_2_ Laves phase was stable in the absence or presence of Hf in the alloy (e.g., alloys ZX4 and ZX8 and EZ6 and EZ8 in Table 1). With the addition of Fe, FeNb_4_Si (τ_4_ phase) was stable in alloys NV3 and NV5, and Fe_7_Nb_6_ (μ phase) and the π phase were also formed in NV5–AC [22]. In NV2, the τ_4_ was suppressed with the additions of Al and Hf, the μ phase was observed in the cast microstructure and only the π phase was observed in NV2–HT1 and NV2–HT2. The <Si> =Si + Al + Sn = 11.1 at.% of the μ phase was within the range reported by Wang et al. [21]. The π phase was formed where the μ phase was observed in the cast microstructure, i.e., it is likely that Fe_7_Nb_6_ transformed into the π phase. The point at issue is whether the compound that was designated as the π phase is a Laves phase. 

In the Fe–Nb binary, the μ phase and the C14–NbFe_2_ Laves (ε) phase are in equilibrium [50]. In the Nb–Fe–Si ternary, there is three-phase equilibria between ε, μ and αNb_5_Si_3_ at 1000 and 1200 °C [21]. Comparison with the alloys NV3 and NV5 would suggest that the addition of Al and Hf destabilised the τ_4_ phase and favoured the μ phase in NV2. The latter was present everywhere in the microstructure of NV2–AC, unlike NV5–AC, where its formation was sensitive to solidification conditions and was suppressed at the high cooling rates prevailing in the bottom of the ingot. Compared with NV5–AC, in NV2–AC, the solubility of Cr in the μ phase increased significantly at the expense of Ti and Fe. 

**Figure 14:**
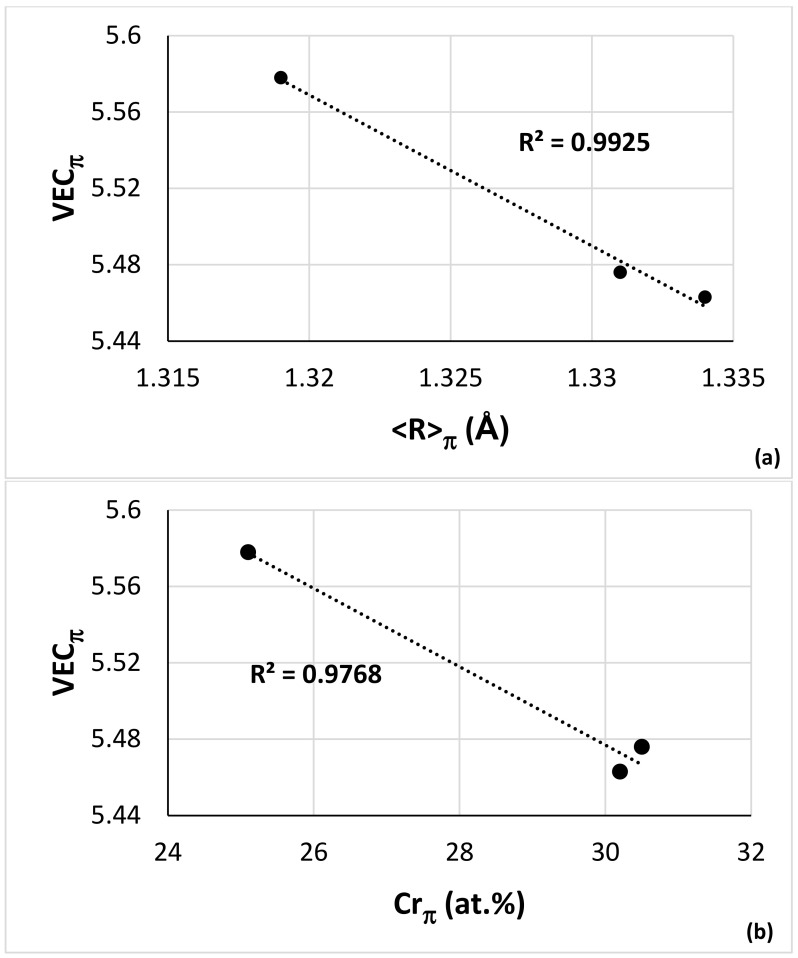
(**a**) VEC versus <R> and (**b**) VEC versus Cr of the π phase in the alloys NV2 and NV5. The parameter VEC was calculated as described in [51]. <R> = R _<A>_ + R_<B>_, where R_<A>_ = ∑_i_^n^C_i_(r_<A>_)_i_, C_i_ and (r_<A>_)_i_, respectively, are the concentration (at. %) and atomic radius of Nb and element i substituting Nb in the π phase, and R_<B>_ = ∑_i_^n^C_i_(r_<B>_)_i_ is the concentration (at.%) and atomic radius of Cr and element i substituting Cr in the π phase. For A and B, see the text.

If we present the C14-NbCr_2_ Laves phase in Fe-free RM(Nb)ICs as AB_2_, where A = Nb; Ti and Hf and B = Cr and Al, Ge, Si and Sn, then the data show 50.9 < B < 62 at.% for as-cast alloys and 53.1 < B< 64.5 at.% for heat-treated alloys [51] compared with 61.8 < Cr < 69.5 at.% in the C14–NbCr_2_ in the Nb–Cr binary [50]. In other words, the alloying of the C14–NbCr_2_ Laves phase shifts B to lower values. For the ε phase, 62.7 < Fe < 65.9 at.% [50] in the Nb–Fe binary, whereas, according to the data of Wang et al. [21] for the C14 Nb(Fe,Si)_2_ (ε) Laves phase in the Nb–Fe–Si ternary, the Si solubility was substantial (<27.7 at.%), and the B (=Fe, Si) was in the range 63.8 < B < 76 at.% and 64.6 < B < 75.3 at.%, respectively, at 1000 and 1200 °C. In other words, the alloying of ε in the ternary moved B to higher values. The solubility of Si in the π phase was less than 11 at.% in NV5 and NV2, and B(=Cr + Fe + Al + Si + Sn) was 58 at.% in NV5–AC, 56.6 at.% in NV2–HT1 and 58 at.% in NV2–HT2, i.e., it was in the range of the B content for the C14 Laves phase in Fe-free RM(Nb)ICs (A = Nb, Ti and Hf in the π phase). Bearing in mind that we have only three data points, the trends in the VEC_π_ versus <R > _π_ plot (Figure 14a) and <R> _A,π_/< R>_B,π_ versus <R> _π_ plot (figure not shown) were the same with those of the C14–NbCr_2_-based Laves phase in Fe-free RM(Nb)ICs [51] but not the trend in the VEC_π_ versus Cr_π_ plot (Figure 14b). Given that the XRD provided no conclusive evidence for the presence of a C14 Laves phase in NV2–HT1 and NV2–HT2, we cannot confirm that the π phase is a C14 Laves phase.

Oxides of Hf and/or Ti were not observed in NV2–AC, even though the latter was formed in the alloys NV3, NV5 [22] and NV8 [31] and the former in NV1–AC [32], which would suggest that the synergy of Fe with Al and Hf had some effect regarding the “control” of the contamination of the microstructure of NV2 with oxygen.

### 4.2. Hardness and Specific Strength

The hardness of the heat-treated alloy NV2 increased compared with NV2–AC. The increase of the hardness of NV2–HT1 was attributed to the increase and decrease of the volume fractions of A15–Nb_3_X and Nb_ss_, respectively (Table 2). The decrease of the hardness of NV2–HT2 was attributed to the decrease of the volume fraction of A15–Nb_3_X compared to NV2–HT1 (Table 2).

**Figure 15:**
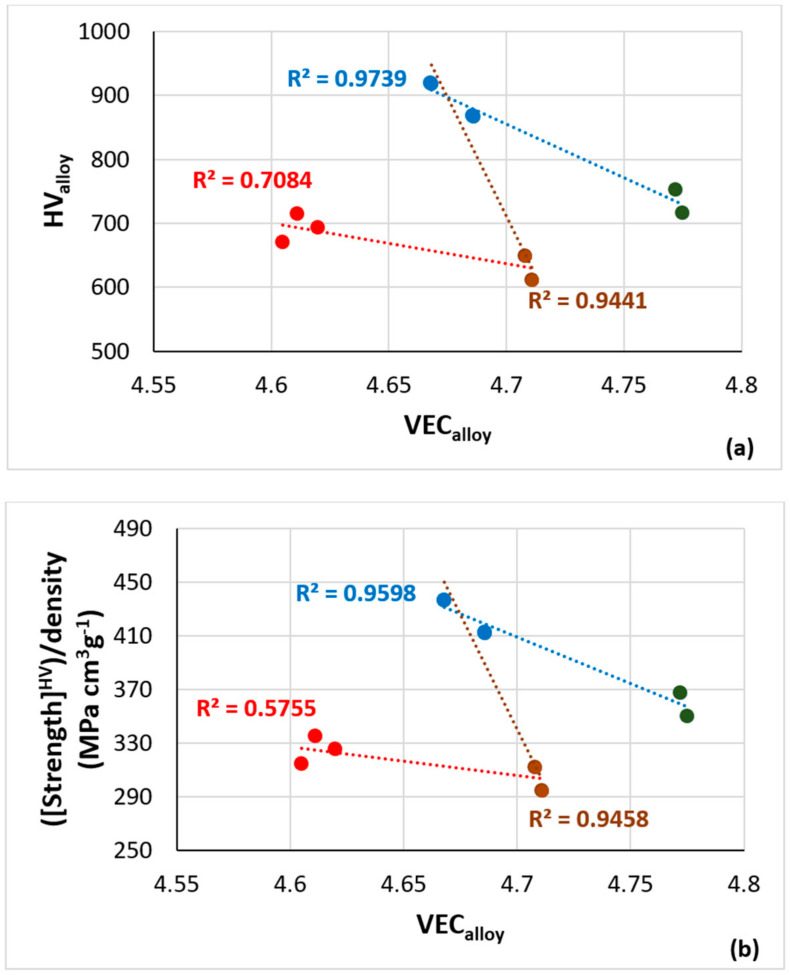

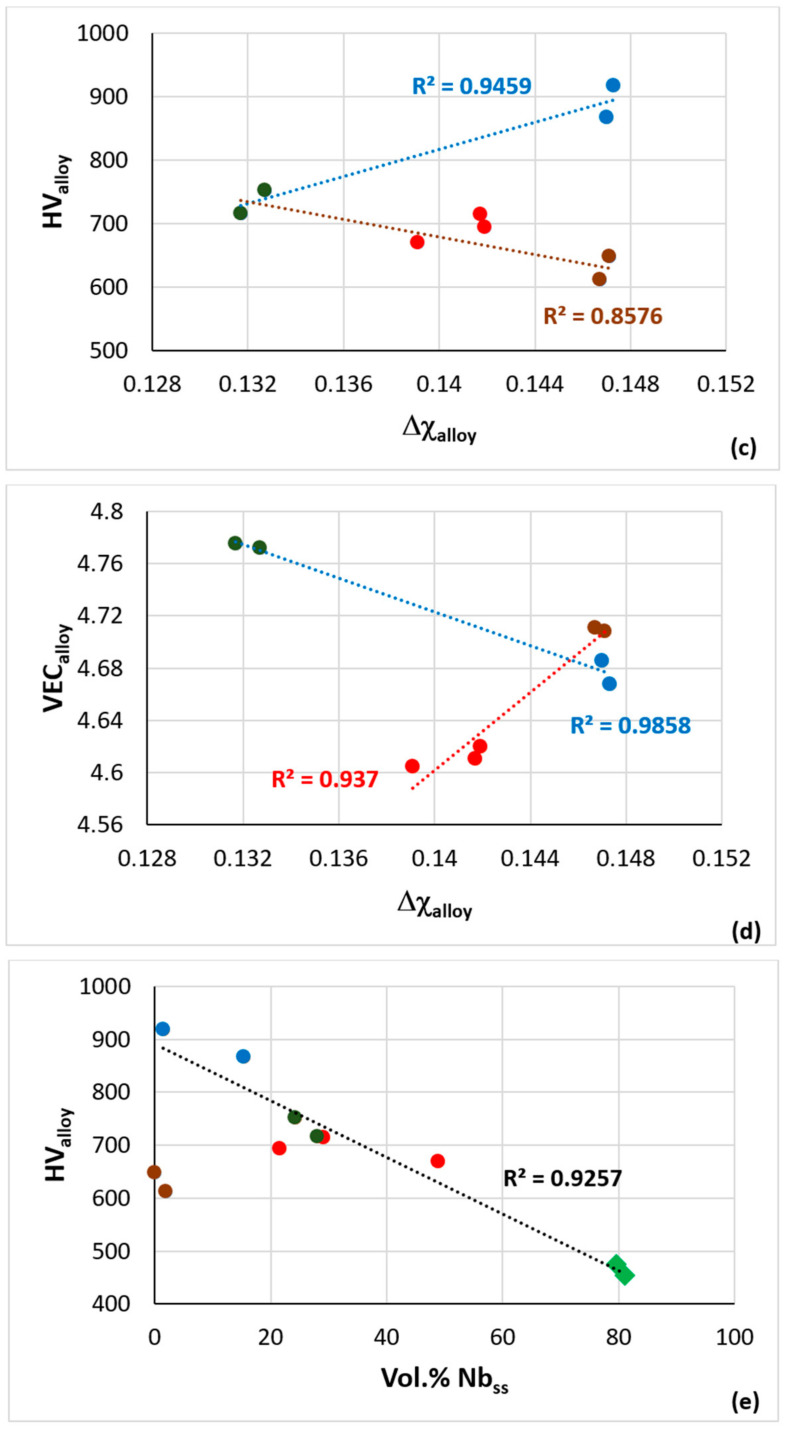
(**a**) Alloy hardness versus VEC, (**b**) specific strength calculated from hardness versus VEC, (**c**) alloy hardness versus parameter Δχ, (**d**) VEC versus Δχ and (**e**) alloy hardness versus vol.% Nb_ss_. Colours: (**a**–**e**) Red NV2, blue NV8, green NV3 and brown NV5. In (**e**), light green diamonds for alloy NV1 [32].

The hardness of the Fe-containing alloys NV2, NV3, NV5 and NV8 was plotted versus the parameters VEC and Δχ in Figure 15a,c, and the room temperature-specific strength calculated from the hardness was plotted versus VEC in Figure 15b. Changes of the parameters VEC and Δχ with alloying additions are shown in Figure 15d. The substitution of Cr (in NV3) with Sn in NV8 decreased and increased VEC and Δχ, respectively, while the addition of Cr in NV5 increased and deceased VEC and Δχ, respectively. Alloy NV2 had lower values of VEC and Δχ, compared with NV5. The alloy hardness decreased as the vol.% Nb_ss_ increased, but the data for NV5 did not fall on the linear fit with R^2^ = 0.9257 of the data for the Fe-containing alloys NV2, NV3 and NV8 and the Fe-free alloy NV1 (Figure 15e).

The data for the alloys NV3 and NV8 showed that, as the Cr (in NV3) was substituted with Sn in NV8, the hardness increased as the parameters VEC and Δχ, respectively, decreased and increased. The data for the alloys NV8 and NV5 showed that, with the addition of Cr in NV5 (the alloy NV5 actually is NV8 plus 5 at.% Cr), the hardness decreased as the parameter VEC increased (Figure 15a), whereas the parameter Δχ did not change significantly (Figure 15c). (In Figure 15c, the linear fit of the data for NV8 and NV5 gives R^2^ = 0.4899, the linear fit not shown). 

The data for alloys NV5 and NV2 in Figure 15a,c also show the increase of the hardness of NV2 with the decrease of the parameters VEC and Δχ. Furthermore, the data for alloys NV3 and NV5 in Figure 15c (the alloy NV5 actually is NV3 plus 5 at.% Sn) show that, with the addition of Sn, the hardness was reduced, and the parameter Δχ increased (R^2^ = 0.8576 for the linear fit) and that the data for alloy NV2 falls on the same trend. The specific strength of NV2 was comparable with that of the RCCAs reviewed in [4] and RM(Nb)ICs, e.g., see [5,7,30], whereas that of alloy NV8 (where Fe was in synergy with Sn but without Al, Cr and Hf additions) was higher and/or comparable with that of Fe-free RM(Nb)ICs and RM(Nb)ICs/RCCAs [5,7,25,30].

### 4.3. Oxidation 

The oxidation of NV2 at 700 °C was better than the other NV series alloys and the MASC alloy, even better than alloys NV5 and NV8 (Figure 4a), which had lower vol.% Nb_ss_ and not stable Nb_ss_ after the heat treatment [22,31], and better than the alloy NV1, with significantly higher vol.% Nb_ss_ (about 80%) [32]. The parabolic rate constant of NV2 was one order of magnitude lower than that of the alloys NV5 and NV8 and two orders of magnitude lower than the Sn-free alloy NV3 (Table 4). The data would suggest that the synergy of Fe with Al, Cr, Hf and Sn (NV2) was better than that with Cr and Sn (NV5) regarding oxidation at 700 °C and 900 °C (Figure 4a,c), but the opposite was the case at 800 °C where the mass change of NV2 was slightly higher than that of NV5 (Figure 4b), and its parabolic rate constant was one order of magnitude higher than that of NV5 and similar to that of NV8 (Table 4). Furthermore, at 900 °C, the mass change of NV2 was higher than that of alloys NV1 and NV3, even though the volume fraction of Nb_ss_ was about 60% lower or higher than both alloys, and alloy NV3 did not have a Sn addition. Additionally, the data in Table 4 would suggest (i) that the addition of Sn was not as effective at 900 °C as at 700 and 800 °C and (ii) that Sn was very effective at 700 °C without other elements that are known to improve the oxidation of RM(Nb)ICs and RM(Nb)ICs/RCCAs, meaning the case of alloy NV6. 

**Figure 16:**
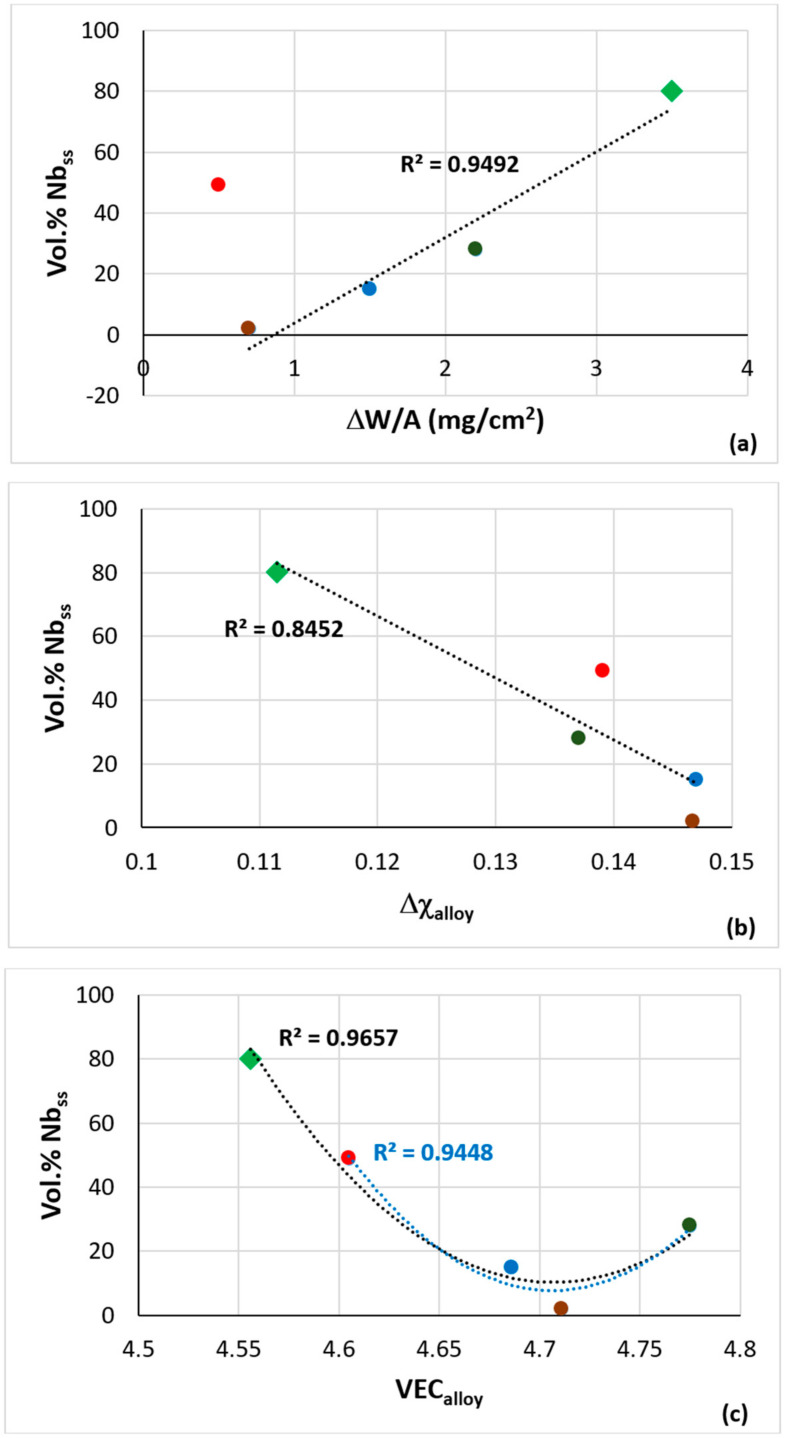

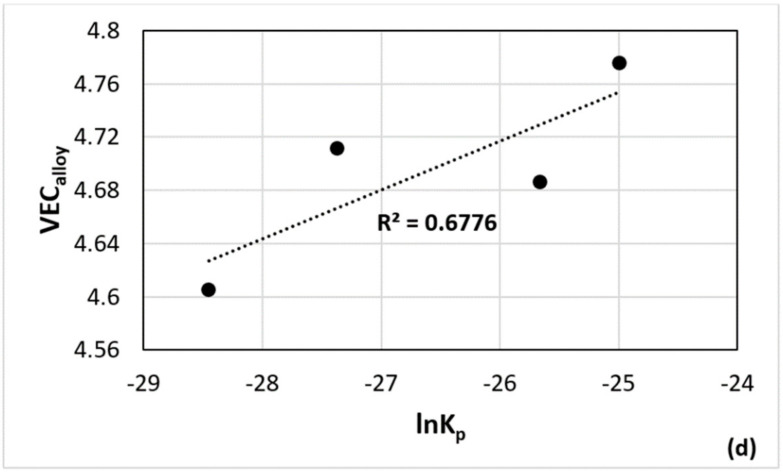
(**a**) Vol.% Nb_ss_ versus the mass change ΔW/A at 700 °C, (**b**) vol.% Nb_ss_ versus parameter Δχ, (**c**) vol.% Nb_ss_ versus VEC and (**d**) VEC versus lnK_p_ at 700 °C. Colours: red is NV2, blue is NV8, green is NV3, brown is NV5 and light green diamonds for alloy NV1.

Nb_ss_ plays an important role in the oxidation of RM(Nb)ICs and RM(Nb)ICs/RCCAs or RM(Nb)ICs/RHEAs. It is thought that the mass change (ΔW/A) of these metallic UHTMs increases as the vol.% Nb_ss_ increases. Alloy NV1 demonstrated (i) that this is not always the case and (ii) that, as one would expect, the chemical composition is also important [32]. Indeed, for isothermal oxidation at 700 °C, the mass change increased with vol.% Nb_ss_ (Figure 16a), but the data of the alloy NV2 did not fall during the trend specified by the data for the other Fe-containing alloys and alloy NV1. Furthermore, for the Fe-containing alloys and alloy NV1, the vol.% Nb_ss_ increased as the parameters Δχ_alloy_ or VEC_alloy_ decreased (Figure 16b,c), whereas the mass change of the Fe-containing alloys that followed parabolic oxidation kinetics at 700 °C decreased with the decreasing VEC, in agreement with NICE [9], as did the parabolic rate constant (Figure 16d). Note that, in Figure 16c, the linear fit of all the data was R^2^ = 0.6308, and the parabolic fit was R^2^ = 0.9657, with the minimum corresponding to VEC = 4.725 and vol.% Nb_ss_ = 10%, whereas, for the Fe-containing alloys, i.e., excluding alloy NV1, the parabolic fit was R^2^ = 0.9448, with the minimum at VEC = 4.723 and vol.% Nb_ss_ = 10%.

The microstructure near the scale/substrate interface and the bulk of RM(Nb)ICs and RM(Nb)ICs/RCCAs after exposure to high temperatures is key to understanding the performance of these metallic UHTMs in structural applications, especially given that refractory metals and their alloys can be contaminated with interstitials, and environmental coatings must protect the substrate from interstitial contamination [5,6,7,9]. The microstructure of alloys NV2 and NV5 after isothermal oxidation at 800 °C is compared in Table 9 and Table 10. 

**Figure 17:**
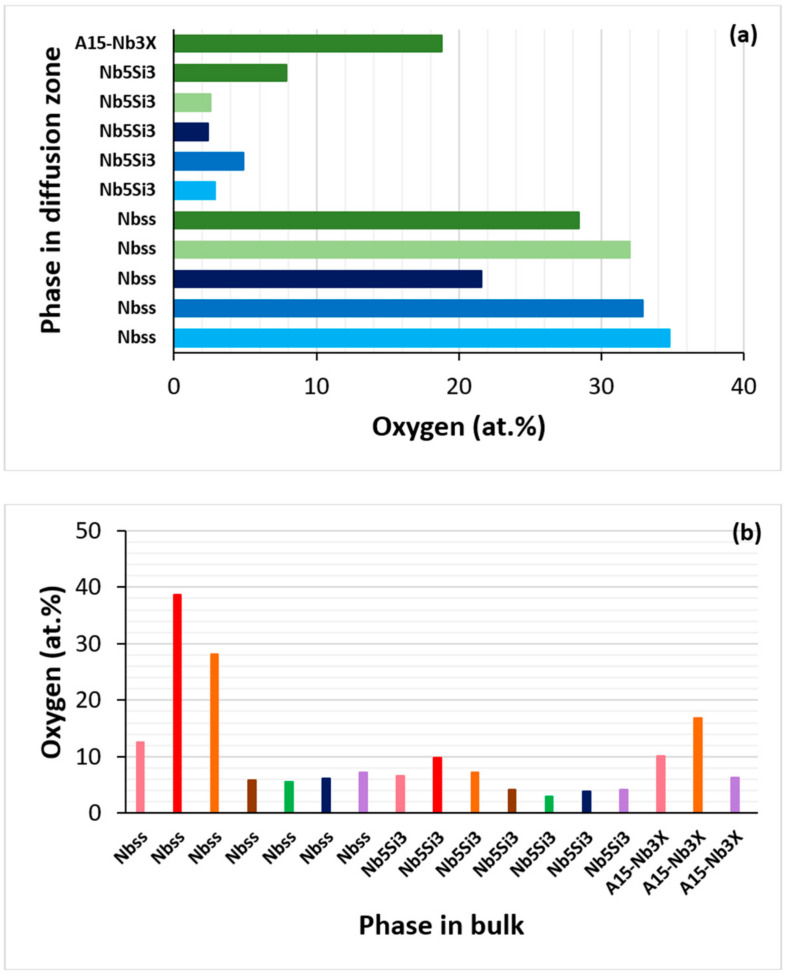
Contamination of the phases with oxygen after isothermal oxidation at 800 °C (**a**) in the diffusion zone (DZ) and (**b**) in the bulk. In (**a**), Nb_ss_ from bottom to top in alloys ZX5, ZX7, ZF4, ZF5 [26] and NV2; Nb_5_Si_3_ from bottom to top in alloys ZX5, ZX7, ZF4, ZF5 [26] and NV2 and A15–Nb_3_X in NV2. In (**b**), Nb_ss_ from left to right in alloys NV2, NV1, NV5, ZX3, ZX5, ZX7 and ZX4; Nb_5_Si_3_ from left to right in alloys NV2, NV1, NV5, ZX3, ZX5, ZX7 and ZX4 and A15–Nb_3_X from left to right in alloys NV2, NV5 and ZX4. Same colour is used for each alloy in (**a**) or in (**b**). All analysis data was obtained using EPMA with standards.

The oxidation performance of NV2 in the three pest oxidation temperatures was significantly better than that of the RHEAs or RCCAs, even those that were rich in Al content [4,52]. The contamination of the phases in the diffusion zone and in the bulk is compared in Figure 17. Note that, in this figure, the phases in alloys NV2, NV5 or NV1 are compared with phases in other Fe-free but Sn or Ge-containing RM(Nb)ICs. In the diffusion zone (DZ) in NV2, Nb_ss_ was more severely contaminated than Nb_5_Si_3_, while the contamination of A15–Nb_3_X was in between that of Nb_ss_ and Nb_5_Si_3_. Furthermore, the contamination of Nb_ss_ and Nb_5_Si_3_, respectively, was less and more severe compared with the 2 at.% Sn-containing alloys ZX5 and ZX7 (see Table 1 and Table 8 for the nominal compositions) [27]. Compared with alloy ZX8 with a 5 at.% Sn addition, the contamination of Nb_5_Si_3_ and A15–Nb_3_X was more severe in NV2 [28]. The lowest contamination of Nb_ss_ and Nb_5_Si_3_ in the DZ was for the Ge-containing alloy ZF4 (Nb–24Ti–18Si–5Cr–5Ge [26]).

In the bulk, the contamination of Nb_ss_ was more severe than that of Nb_5_Si_3_, and the contamination of A15–Nb_3_X was in between Nb_ss_ and Nb_5_Si_3_. The contamination of Nb_ss_ or Nb_5_Si_3_ increased from NV2 to NV5 to NV1 and of A15–Nb_3_X from NV2 to NV5. In alloys NV2, NV5 and NV1, the contamination of Nb_ss_, Nb_5_Si_3_ and A15–Nb_3_X was more severe than in alloys ZX3, ZX5, ZX7 and ZX4 (see Table 8 for the nominal compositions). Compared with alloy ZX7, the contamination of Nb_ss_ and Nb_5_Si_3_ in NV2 was almost twice higher. Compared with alloy ZX4, the contamination of A15–Nb_3_X in NV2 was 1.5× higher, whereas, compared with alloy ZX8, the contamination of Nb_5_Si_3_ and A15–Nb_3_X in NV2 was 1.7× and 1.6× higher, respectively. 

The most severe contamination of Nb_ss_ and Nb_5_Si_3_ in the bulk of oxidised alloys was in the phases alloyed with V in alloy NV1 [32]. The lowest contamination of Nb_ss_ and Nb_5_Si_3_ in the bulk of oxidised alloys was in the phases alloyed with Al and Sn in the 2 at.% Sn alloy ZX5. The lowest contamination of A15–Nb_3_X in the bulk of oxidised alloys was in the phase alloyed with Cr and Sn in the 5 at.% Sn alloy ZX4.

The contamination of the π phase in the DZ of NV2 was about three times higher than in the bulk. In the bulk of NV5, the contamination of the τ_4_, μ and Nb_3_Si-m’ compounds was slightly more severe than the π phase in the bulk of NV2 (Table 5 and Table 6). 

### 4.4. CC and HE Phases

Often, in as-cast and/or heat-treated RM(Nb)ICs and RM(Nb)ICs/RCCAs or RM(Nb)ICs/RHEAs, “conventional” phases coexist (form together) with complex concentrated (CC) phases [7] (also referred to as compositionally complex (CC) phases) or high entropy (HE) phases [7,9], depending on their chemical composition, where the concentration of elements either complies with the “standard definition” of HEAs or the higher or lower concentration of the elements is above or below 35 and 5 at.%, i.e., the upper and lower limits in the “standard definition” [4]. In Reference [7], these phases were designated HE phases, when the actual concentrations of the elements were in the range from 35 to 5 at.% and the CC phases when the actual concentrations of the elements was >35 and <5 at.%. Examples of such phases—namely, alloyed Nb_ss_, Nb_5_Si_3_, A15–Nb_3_X, C14–NbCr_2_ Laves and eutectic with Nb_ss_ and Nb_5_Si_3_ in as-cast and/or heat-treated RM(Nb)ICs and RM(Nb)ICs/RCCAs—can be found in Table 1 in [7]. The CC phases in the as-cast and heat-treated alloy NV2 are summarised in Table 11. 

In the NV2–AC CC phases were the Nb_5_Si_3_ and tP32–Nb_3_Si silicides, the μ phase and the Nb_ss_ + Nb_5_Si_3_ eutectic. After the heat treatments, the CC phases were the Nb_5_Si_3_, tP32–Nb_3_Si and Nb_3_Si-m’ silicides and the π phase. Additionally, the CC phases formed after oxidation at a pest temperature in the diffusion zone (DZ) and/or in the bulk of oxidised alloy NV2 (Table 11). Indeed, CC Nb_5_Si_3_ and the π phase and CC Nb_ss_ were formed in the DZ of NV2 and CC Nb_5_Si_3_ and the π phase in the bulk of NV2 after the isothermal oxidation at 800 °C. Furthermore, after the isothermal oxidation at 800 °C, in the bulk of NV5, the CC phases were Nb_3_Si-m’ and the solid solution, and the HE phases were τ_4_ and μ. Note that, in the bulk of oxidised alloy NV1, there was CC Nb_5_Si_3_, CC Nb_ss_ + Nb_5_Si_3_ eutectic and CC Nb_ss_ and Ti-rich Nb_ss_ [32] (see Table 11).

The data for alloys NV1, NV2 and NV5 confirmed that CC and/or HE phases are formed in Sn-containing RM(Nb)ICs with/without Fe addition not only in the cast and/or heat-treated microstructures but also after oxidation at a pest temperature or at a higher temperature. This is supported by the data for specific phases in the DZ of alloys ZX5, ZX7 [27] and ZX8 [28] at 800 °C (CC phases Nb_ss_ and Nb_5_Si_3_ in [27] and CC Nb_ss_+Nb_5_Si_3_ eutectic in [28]) but also for oxidation at 1200 °C. Indeed, CC NbSn_2_ [27,28] and Nb_5_Si_3_ [27] formed in the Sn-rich zone below the oxide scale at 1200 °C, respectively, in ZX3 [27] and ZX4 [28] and in ZX5 [27] and CC C14–NbCr_2_ Laves phase in the bulk of ZX7 [27]. 

The data for the aforementioned CC and HE phases in Sn-containing RM(Nb)ICs in the as-cast, heat-treated and oxidised conditions at a pest or higher temperature is in a new database in NICE for CC or HE phases in metallic UHTMs with Nb and Si additions (see Table 1 in [7]). This database, together with the Fe database that is built up with the data in this paper and in [22,31], is used to design metallic UHTMs with a balance of properties, utilising the design approach outlined in [5,7,9].

### 4.5. Comparison of Experimental Data with NICE

The measured mass change of NV2 at 800 °C was 3.5 mg/cm^2^, and the mass change calculated using NICE and the actual composition of NV2–AC was 3.7 mg/cm^2^ compared with the property target of 5 mg/cm^2^ that was used for the design of the alloy (see Introduction). The constraint (ii) was met as the hardness of the alloy was higher than 600 HV. Additionally, the constraint (iii) was realised for NV2–HT1 and NV2–HT2, where the ratio (vol.% A15–Nb_3_X)/(vol.% Nb_ss_) was greater than one.

## 5. Conclusions

In this work, alloy Nb–30Ti–10Si–5Cr–5Sn–3Fe–2Al–2Hf (NV2) was studied in the as-cast and heat-treated conditions: its isothermal oxidation at three pest oxidation temperatures and its room temperature hardness and specific strength were evaluated and compared with other Sn-containing RM(Nb)ICs—in particular, alloy NV5—and with RCCAs and RHEAs. The issues that motivated this research were resolved. The synergy of Fe with Al, Cr, Hf and Sn (a) stabilised Nb_ss_; A15–Nb_3_X (X = Al, Si and Sn) and Nb_3_Si, metastable Nb_3_Si-m’ and Nb_5_Si_3_ silicides; (b) supported the formation of Nb_ss_ + Nb_5_Si_3_ eutectic in the cast microstructure; (c) suppressed pest oxidation in the temperature range 700–900 °C and (d) stabilised a Cr-rich π phase instead of a C14–Nb(Cr,Fe)_2_ Laves phase. Furthermore, it was shown that complex concentrated (CC) or high entropy (HE) phases coexisted with “conventional” phases in all conditions and after oxidation at 800 °C. 

The alloying with Fe reduced the macrosegregation of Si but necessitated heat treatments at T ≤ 1200 °C owing to liquation at higher temperatures. A solid solution free of Si and rich in Cr and Ti was stable after the heat treatments, and its parameter δ was less than 5. Relationships between solutes in the various phases, between solutes and alloy parameters and between alloy hardness or specific strength and alloy parameters were established. The new data presented in this paper made possible the creation of a Fe database, as well as enlargement of the database for the CC and HE phases in NICE for RM(Nb)ICs, RM(Nb)ICs/RCCAs, RM(Nb)ICs/RHEAs and RCCAs and RHEAs with Nb and Si additions. The specific strength of NV2 was comparable to that of RCCAs and RHEAs that were reviewed in [4], and its oxidation at all three temperatures was significantly better than RHEAs and RCCAs. 

The oxidation of NV2 at 700 °C was better than the other NV series alloys and the MASC alloy, even better than NV series alloys with lower vol.% Nb_ss_. At 800 °C, the mass change of NV2 was slightly higher than that of NV5. In NV2 and NV5, a more or less continuous layer of Nb_3_Sn was formed at the interface with the scale at 800 °C, but in NV5, this Nb_3_Sn was Sn-rich and severely oxidised. At 800 °C, in the diffusion zone (DZ) and the bulk of NV2, Nb_ss_ was more severely contaminated with oxygen than Nb_5_Si_3_, and the contamination ofA15–Nb_3_X was in between that of Nb_ss_ and Nb_5_Si_3_. The contamination of all three phases was more severe in the DZ. The contamination of Nb_ss_, Nb_5_Si_3_ and A15–Nb_3_X in the bulk of NV5 was more severe compared with NV2.

## Figures and Tables

**Table 1:** Stable phases in heat-treated Nb-18Si-based RM(Nb)ICs (at.%) with additions of Al, Cr, Fe, Hf, Sn or Ti, where NV9 = Nb-18Si-5Sn, NV6 = Nb-24Ti-18Si-5Sn, KZ4 = Nb-24Ti-18Si-5Cr, KZ7 = Nb-24Ti-18Si-5Al, KZ5 = Nb-24Ti-18Si-5Al-5Cr. A15-Nb_3_X (X = Al, Si, Sn), metastable 3-1 silicides Nb_3_Si-m and Nb_3_Si-m’ [11]. Note that EZ8 is an RM(Nb)IC/RCCA alloy. (X = phase is present in alloy).

Alloy	Nb_ss_	A15-Nb_3_X	Nb_5_Si_3_	tP32 Nb_3_Si	Nb_3_Si-m	Nb_3_Si-m’	C14-NbCr_2_	FeNb_4_Si	Ref.
NV9	X	X	X	-	-	-	-	-	[22]
NV6	X	X	X	-	-	-	-	-	[22]
EZ2 = NV6 + 5Hf	X	X	X	-	-	-	-	-	[30]
NV8 = NV6 + 5Fe	-	X	-	X	-	-	-	-	[31]
ZX4 = NV6 + 5Cr	X	X	X	-	-	-	X	-	[28]
ZX6 = NV6 + 5Al	-	X	X	-	-	-	-	-	[28]
ZX8 = NV6 + 5Al + 5Cr	X	X	X	-	-	-	X	-	[28]
EZ5 = EZ2 + 5Al	-	X	X	-	-	-	-	-	[30]
EZ6 = EZ2 + 5Cr	X	X	X	-	-	-	X	-	[30]
EZ8 = EZ2 + 5Al + 5Cr	-	X	X	-	-	-	X	-	[30]
KZ4	X	-	X	-	-	-	-	-	[15]
NV3 = KZ4 + 5Fe	X	-	X	-	X	X	-	X	[22]
NV5 = KZ4 + 5Fe + 5Sn	-	X	X	-	-	X	-	X	[22]
ZX3 = KZ4 + 2Sn	X	-	X	-	-	-	X	-	[27]
KZ7	X	-	X	-	-	-	-	-	[15]
ZX5 = KZ7 + 2Sn	X	X	X	-	-	-	-	-	[27]
KZ5	X	-	X	-	-	-	-	-	[15]
ZX7 = KZ5 + 2Sn	X	X	X	-	-	-	X	-	[27]

**Table 2:** Density (*ρ*), Vickers hardness (HV) and %area of Nb_ss_ and A15–Nb_3_X in the alloy NV2.

Condition	*ρ* (g/cm^3^)	Hardness (HV)	% Area of Nb_ss_	% Area of Nb_3_X
NV2–AC	6.93–7.06	606–724	46.6–54.4	14.8–29.5
	6.97 ± 0.1	670 ± 30	48.8 ± 2.6	20.0 ±4.4
NV2–HT1	-	698–762	18.8–42.0	35.1–46.8
	-	715 ± 19	29.1 ± 7.6	40.8 ± 4
NV2–HT2	-	657–715	20.2–22.8	30.7–32.9
	-	694 ± 18	21.5 ± 1.1	31.5 ± 1

**Table 3:** Chemical composition (at.%) of the phases in as-cast and heat-treated alloy NV2.

NV2–AC	Al	Si	Ti	Cr	Fe	Nb	Sn	Hf
Nb_ss_	1.9–2.9	0.8–2.3	27.1–37.0	4.0–6.9	1.3–3.7	43.5–56.0	4.2–5.7	0.0–2.0
	2.5 ± 0.3	1.4 ± 0.3	32.4 ± 2.1	5.8 ± 0.8	2.8 ± 0.7	48.9 ± 3	4.9 ± 0.4	1.3 ± 0.5
Ti-rich Nb_ss_	2.4–3.2	0.7–2.0	37.2–50.2	3.6–5.9	2.9–5.3	28.7–44.0	4.5–6.0	1.3–2.5
	2.7 ± 0.2	1.0 ± 0.2	43.0 ± 3.2	4.9 ± 0.7	4.2 ± 0.6	37.4 ± 3.8	5.1 ± 0.4	1.7 ± 0.4
A15-Nb_3_X	1.8–2.9	4.1–6.7	21.9–30.2	1.5–3.4	0.1–1.8	46.7–57.9	9.7–12.0	0.0–1.6
	2.4 ± 0.2	5.1 ± 0.5	25.4 ± 2.3	2.3 ± 0.5	0.7 ± 0.4	52.9 ± 3	10.9 ± 0.7	0.3
Nb_5_Si_3_	1.3–1.9	29.5–34.6	22.1–30.8	0.8–1.8	0.2–2.9	26.1–40.0	0.4–2.4	1.2–4.8
	1.5 ± 0.2	31.9 ± 1.3	26.5 ± 2.6	1.2 ± 0.2	1.5 ± 0.7	33.1 ± 3.9	1.5 ± 0.6	2.8 ± 1.1
Ti-lean Nb_5_Si_3_	0.6–1.1	34.6–36.1	18.4–20.9	0.1–0.7	0.2–0.7	37.5–40.9	0.9–1.4	2.5–3.3
	0.8 ± 0.2	35.3 ± 0.4	19.9 ± 0.7	0.2	0.3	39.5 ± 1	1.2 ± 0.1	2.8 ± 0.3
Hf-rich Nb_5_Si_3_	1.1–1.8	34.3–36.0	26.8–30.7	0.6–1.8	0.2–1.2	22.7–30.8	0.1–0.7	3.4–7.4
	1.5 ± 0.2	35.2 ± 0.5	28.4 ± 1.1	1.0 ± 0.3	0.7 ± 0.4	27.9 ± 2	0.2	5.1 ± 1
Fe_7_Nb_6_ μ phase	1.5–2.0	8.0–9.8	20.2–25.5	20.6–28.1	17.1–21.5	16.2–22.3	0.0–0.8	2.6–5.5
	1.8 ± 0.2	9.1 ± 0.4	23.0 ± 1.4	24.2 ± 1.9	19.0 ± 1.4	18.8 ± 1.5	0.2	3.9 ± 0.8
Nb_3_Si	1.0–2.1	22.6–24.6	23.3–31.5	2.0–4.8	1.8–3.6	29.9–42.5	0.5–3.5	2.6–4.0
	1.7 ± 0.5	23.5 ± 0.8	29.2 ± 3.9	2.6 ± 1.5	2.6 ± 0.9	34.8 ± 5.4	2.2 ± 1.4	3.4 ± 0.6
Nb_3_Si-m’	0.6–0.9	26.7–27.7	21.3–22.8	0.6–1.7	3.6–4.5	41.2–43.1	-	2.2–3.2
	0.8 ± 0.2	27.2 ± 0.3	22 ± 0.5	1 ± 0.3	4 ± 0.3	42.1 ± 0.6	-	2.9 ± 0.4
Eutectic	1.6–2.2	14.1–19.4	26.4–31.9	3.2–4.1	1.5–3.6	36.7–42.0	2.3–3.1	2.2–3.4
	1.9 ± 0.2	16.7 ± 1.6	31.1 ± 1.4	3.7 ± 0.3	2.4 ± 0.6	38.7 ± 1.1	2.9 ± 0.4	2.6 ± 0.4
**NV2–HT1**								
Nb_ss_ no Si	1.5–2.2	-	33.3–37.1	3.6–4.7	1–2.7	50.1–55.4	2.6–3.3	0.8–1.3
	2 ± 0.2	-	34.8 ± 1.3	3.9 ± 0.5	1.9 ± 0.8	53.5 ± 2	2.9 ± 0.3	1 ± 0.2
Nb_ss_	2.4–2.9	0.5–0.9	37–38.6	4.2–5.3	1.7–2.1	46.7–48.1	3.3–4	1.1–1.5
	2.7 ± 0.2	0.7 ± 0.2	37.7 ± 0.6	4.9 ± 0.4	1.8 ± 0.1	47.1 ± 0.3	3.8 ± 0.2	1.3 ± 0.2
A15-Nb_3_X	1.1–4.0	1.4–5.6	19.1–32.0	1.2–4.4	0.2–3.1	42.5–62.0	9.3–14.9	0.4–1.3
	2.7 ± 0.7	3.8 ± 1.2	28.1 ± 3.4	2.1 ± 0.7	1.0 ± 0.8	51.2 ± 4.2	10.4 ± 1.5	0.7 ± 0.2
Nb_5_Si_3_	1.1–1.9	33.1–35.7	27.3–32.7	0.2–2.2	0.3–1.8	23–30.3	0.2–0.7	3.9–5.7
	1.4 ± 0.2	34.8 ± 0.7	30.2 ± 1.4	0.8 ± 0.5	0.9 ± 0.4	26.7 ± 2	0.4 ± 0.2	4.8 ± 0.5
Si-lean Nb_5_Si_3_	1.4–1.5	30.7–33	29.2–34.7	1.2–4.2	0.8–3.0	21.8–28.9	0.4–0.8	4.2–5.0
	1.5 ± 0.1	31.8 ± 1.2	30.8 ± 2.6	2.7 ± 1.4	2.0 ± 1	26.0 ± 3.1	0.6 ± 0.2	4.6 ± 0.3
π phase	1.2–1.7	6.9–11.5	20.4–22.5	23.9–32.3	13.5–18.7	16.2–22.8	-	3.5–4.4
	1.4 ± 0.2	7.6 ± 0.3	21.4 ± 0.4	30.3 ± 0.8	17.3 ± 0.5	18.1 ± 0.6	-	3.9 ± 0.2
Nb_3_Si	0.9–1.1	23.9–24.1	25.6–25.9	1.2–4.1	4.4–5.5	36–39.7	0.1–0.6	3.5–3.6
	1.0 ± 0.1	24.0 ± 0.1	25.7 ± 0.2	2.7 ± 2.1	4.9 ± 0.8	37.8 ± 1.2	0.3	3.6 ± 0.1
Nb_3_Si-m’	0.5–1.0	25.7–28.7	22.8–26.9	0.7–4.0	3.0–5.4	35.0–41.0	0.1–0.7	2.9–3.6
	0.7 ± 0.2	26.9 ± 0.8	25.6 ± 0.8	1.8 ± 0.8	4.3 ± 0.6	37.2 ± 1.3	0.2	3.3 ± 0.2
**NV2–HT2**								
Nb_ss_ no Si	2.4–3	–	37.3–41.4	4.1–5.8	1.3–2.6	43.9–49	3.2–4.6	1.1–1.5
	2.7 ± 0.2	–	38.7 ± 1.2	5 ± 0.7	1.9 ± 0.5	46.6 ± 2.2	3.8 ± 0.6	1.3 ± 0.2
Nb_ss_	2.5–3.3	0.4–1.2	39–42	5.2–6.6	1.6–2.3	41.1–43.3	3.7–4.6	1.1–1.8
	3 ± 0.3	0.7 ± 0.3	40.7 ± 0.9	6 ± 0.6	1.9 ± 0.3	42.2 ± 0.9	4 ± 0.2	1.5 ± 0.3
A15-Nb_3_X	2.8–3.8	2.5–4.2	31–33.5	2.3–3.3	0.2–1.4	42.8–46.8	11.3–13.0	0.7–1.0
	3.3 ± 0.3	3.1 ± 0.4	32.1 ± 0.8	2.7 ± 0.2	0.8 ± 0.5	45.2 ± 1.1	12.0 ± 0.6	0.8 ± 0.1
Hf-rich Nb_5_Si_3_	1.2–1.7	35.1–36.1	26.9–31.6	0.2–0.7	0.6–1.0	22.2–30.4	0.1–0.5	3.8–6.8
	1.5 ± 0.2	35.7 ± 0.3	28.8 ± 1.7	0.4	0.7 ± 0.1	27.8 ± 3.2	0.3	4.8 ± 1.1
π phase	1.3–2.0	7.5–9.1	18.4–21.4	27.3–32.5	15.9–20.5	17.7–19.5	-	3.6–4
	1.7 ± 0.2	8.3 ± 0.4	19.5 ± 1	30.6 ± 1.9	17.4 ± 1.6	18.7 ± 0.5	-	3.8 ± 0.1
Nb_3_Si	0.8–0.9	23.4–24.4	24.6–26.8	0.9–2.1	2.9–3.9	39.6–40.1	1.5–2.4	2.7–3.1
	0.9 ± 0.1	23.9 ± 1.2	25.6 ± 1.2	1.5 ± 0.6	3.4 ± 0.7	39.9 ± 0.7	1.9 ± 1.2	2.9 ± 0.3
Nb_3_Si-m’	0.2–1.0	27.7–28.9	23.1–24.4	0.6–1.4	3.4–4.3	38–39.4	-	3.2–3.9
	0.6 ± 0.3	28.4 ± 0.3	23.9 ± 0.4	0.8 ± 0.2	3.9 ± 0.3	38.9 ± 0.4	-	3.5 ± 0.2

**Table 4:** Oxidation rate constants of the NV series alloys and the MASC alloy at 700 °C, 800 °C and 900 °C. As-cast specimens were oxidised for all the alloys. Vol.% of Nb_ss_ in the cast alloys are given in [32] and in Table 2.

Alloy	700 °C		800 °C		900 °C
	k_l_ (g·cm^−2^·s^−1^)	k_p_ (g^2^·cm^−4^·s^−1^)	k_l_ (g·cm^−2^·s^−1^)	k_p_ (g^2^·cm^−4^·s^−1^)	k_l_ (g·cm^−2^·s^−1^)
NV1	-	3.4 × 10^−11^	-	2.8 × 10^−10^	5.4 × 10^−8^ (0–16 h), 1.3 × 10^−7^ (>16 h)
NV2	-	4.4 × 10^−13^	-	3.4 × 10^−11^	9.8 × 10^−8^ (0–16 h), 2.0 × 10^−7^ (>16 h)
NV3	-	1.4 × 10^−11^	1.4 × 10^−7^	-	1.4 × 10^−7^
NV5	-	1.3 × 10^−12^	-	1.3 × 10^−12^	3.0 × 10^−7^ (0–55 h), 1.3 × 10^−7^ (>55 h)
NV6	-	1.3 × 10^−11^	3.3 × 10^−8^	-	2.1 × 10^−6^ (0–17 h), 9.3 × 10^−8^ (>17 h)
NV8	-	7.2 × 10^−12^	-	6.1 × 10^−11^	3.1 × 10^−7^ (0–5 h), 9.8 × 10^−7^ (>5 h)
MASC	5.6 × 10^−8^ (>10 h)	6.9 × 10^−12^ (0–10 h)	1.5 × 10^−7^	-	5.3 × 10^−7^

**Table 5:** Chemical composition (at.%) of the phases present in the oxide scale, diffusion zone and the bulk of NV2 after isothermal oxidation at 800 °C for 100 h.

Oxide Scale									
Phase	O	Al	Si	Ti	Cr	Fe	Nb	Sn	Hf
Nb and Ti-rich oxide	69.2–71.2	0.5–1.1	0.5–2.7	5.6–8.3	03–1.3	0.1–0.7	17.3–19.1	0.1–1.1	0.2–0.5
	70.1 ± 0.3	0.8 ± 0.2	1.8 ± 0.4	7.6 ± 0.3	0.8 ± 0.3	0.2	18.1 ± 0.5	0.3	0.3
Si-rich oxide	67.8–69.0	0.3–0.9	9.6–12.7	7.3–10.8	0.1–0.6	0.1–0.6	8.2–9.9	0.1–0.3	1.2–1.8
	68.3 ± 0.4	0.5 ± 0.2	12.0 ± 0.3	8.3 ± 1	0.2	0.1	9.0 ± 0.4	0.1	1.5 ± 0.2
Ti-rich oxide	66.8–68.3	0.3–1.5	0.1–0.4	21.3–28.1	1.0–4.6	0.1–0.4	2.7–6.7	-	0.1–0.3
	67.5 ± 0.2	0.6 ± 0.3	0.2	24.2 ± 1.5	2.6 ± 1	0.2	4.4 ± 1.1	-	0.2
Diffusion zone (analyses up to about 50 μm below oxide scale)									
Nb_ss_	17.5–30.9	1.1–2.3	0.1–1.5	19.2–31.6	2.3–6.1	1.0–3.4	15.2–35.9	2.3–3.9	0.6–1.2
	28.4 ± 2	1.8 ± 0.3	0.8 ± 0.2	24.5 ± 2	4.4 ± 1.1	2.1 ± 0.8	33.8 ± 1	3.2 ± 0.2	1.0 ± 0.1
Nb_5_Si_3_	5.8–14.1	1.2–1.7	27.7–33.6	25.1–28.3	0.5–1.8	0.1–0.9	24.0–29.9	0.1–0.7	3.5–4.4
	7.9 ± 1.1	1.3 ± 0.1	32.2 ± 0.4	26.4 ± 0.5	0.9 ± 0.2	0.4 ± 0.1	26.5 ± 1	0.3	4.1 ± 0.1
A15–Nb_3_X	10.2–36.4	1.5–2.6	2.9–5.5	14.6–28.6	1.1–2.1	0.1–1.4	33.2–48.8	7.6–10.5	0.5–0.8
	18.8 ± 3	2.1 ± 0.2	4.2 ± 0.4	21.3 ± 2.1	1.7 ± 0.3	0.3	41.9 ± 2.1	9.0 ± 0.5	0.7 ± 0.1
Sn-rich A15–Nb_3_X	29.9–33.8	0.1–0.7	1.0–1.5	4.3–12.4	0.1–1.3	0.1–2.9	39.8–46.0	11.2–15.7	0.1–0.6
	31.9 ± 0.8	0.2	1.2 ± 0.1	8.5 ± 0.9	0.7 ± 0.2	1.3 ± 0.8	42.3 ± 1.1	13.5 ± 1.1	0.4
π phase (see text)	7.4–29.8	1.2–2.0	5.5–9.2	14.4–23.9	14.8–24.3	12.4–19.0	15.5–22.2	0.1–2.3	2.2–3.7
	14.6 ± 2.1	1.6 ± 0.3	7.8 ± 0.9	20.0 ± 0.8	19.4 ± 1.3	15.9 ± 1.1	16.9 ± 1.8	0.8 ± 0.4	3.0 ± 0.3
Bulk (analyses from about 50 to about 300 μm below oxide scale)									
Nb_ss_	11.5–14.6	1.6–2.0	0.6–1.4	29.1–34.1	2.5–6.6	1.4–3.4	40.4–44.6	2.1- 3.7	0.7–1.3
	12.6 ± 0.9	1.7 ± 0.3	1.0 ± 0.2	31.7 ± 0.6	4.3 ± 0.8	2.3 ± 0.7	42.8 ± 0.8	2.6 ± 0.2	1.0 ± 0.1
Nb_5_Si_3_	5.8–7.8	1.2–1.3	32.9–33.6	25.6–29.3	0.6–1.0	0.4–1.0	23.3–27.5	0.3–0.4	3.9–4.8
	6.6 ± 0.3	1.2 ± 0.1	33.2 ± 0.2	27.6 ± 0.8	0.8 ± 0.1	0.7 ± 0.2	25.1 ± 0.8	0.4 ± 0.1	4.4 ± 0.2
A15–Nb_3_X	8.0–11.2	1.9–2.4	4.3–6.1	21.0–24.6	1.4–2.1	0.1–0.7	46.8–49.9	9.3–10.4	0.6–0.8
	10.1 ± 0.4	2.2 ± 0.1	5.0 ± 0.6	22.0 ± 0.9	1.8 ± 0.2	0.4	48.2 ± 0.8	9.6 ± 0.3	0.7 ± 0.1
π phase (see text)	4.6–6.0	1.3–1.6	7.8–10.3	21.7–24.8	22.6–25.3	17.4–19.0	15.0–17.0	0.1–0.4	2.9–3.8
	5.4 ± 0.3	1.4 ± 0.1	8.5 ± 0.5	22.9 ± 0.8	23.8 ± 0.8	18.2 ± 0.5	16.3 ± 0.4	0.1	3.4 ± 0.3
Ti oxide (see text)	16.2–20.6	0.9–2.1	1.1–2.4	39.2–44.8	3.1–5.4	7.8–16.4	9.6–18.6	1.2–4.0	1.7–2.8
	18.4 ± 1	1.3 ± 0.4	1.5 ± 0.2	41.1 ± 1	4.3 ± 0.8	12.3 ± 1.2	15.5 ± 1.2	2.2 ± 0.6	2.3 ± 0.4

**Table 6:** Chemical composition (at.%) of the phases present in the oxide scale and the bulk of NV5 after isothermal oxidation at 800 °C for 100 h.

Oxide Scale							
Phase	O	Si	Ti	Cr	Fe	Nb	Sn
Nb and Ti-rich oxide	67.4–69.3	0.9–2.3	7.1–15.6	0.2–2.2	0.1–2.9	14.8–19.5	-
	68.6 ± 0.6	1.6 ± 0.3	10.4 ± 2	1.5 ± 0.5	0.7 ± 0.5	17.2 ± 1.1	-
Si-rich oxide	67.3–69.1	9.7–11.9	4.5–6.5	0.2–1.2	0.1–1.5	13.1–15.8	-
	68.4 ± 0.5	11.0 ± 0.4	5.4 ± 0.5	0.5 ± 0.3	0.4	14.3 ± 0.5	-
Ti-rich oxide	67.4–69.0	0.2–0.9	24.2–27.7	0.8–2.0	0.1–2.5	2.1–4.9	-
	68.1 ± 0.5	0.7 ± 0.2	25.8 ± 0.9	1.4 ± 0.3	0.6	3.4 ± 0.5	-
Nb-rich oxide	66.5–70.0	0.8–2.8	2.5–5.9	0.9–3.2	0.3–0.6	21.6–25.1	-
	67.9 ± 1.1	1.8 ± 0.5	4.6 ± 0.7	2.2 ± 0.8	0.4 ± 0.1	23.1 ± 0.9	-
Mixed oxide (see text)	66.5–68.2	4.8–6.7	4.7–7.9	1.6–3.2	0.4–2.2	14.7–19.5	-
	67.6 ± 0.5	5.7 ± 0.7	6.6 ± 0.9	2.4 ± 0.5	1.0 ± 0.5	16.6 ± 1.1	-
Bulk (analyses up to about 300 μm below oxide scale)							
Nb_ss_ (see text)	11.2–35.8	0.1–0.9	26.7–38.6	1.2–4.2	1.0–5.7	18.4–39.8	3.3–4.5
	28.1 ± 1.9	0.4	34.5 ± 1.9	2.5 ± 0.8	2.6 ± 1	28.1 ± 3	3.8 ± 0.3
Nb_5_Si_3_	5.1–9.8	28.3–31.2	15.6–19.6	0.7–1.4	0.8–2.2	37.9–44.0	1.5–2.1
	7.2 ± 0.8	30.4 ± 0.8	16.4 ± 0.8	1.0 ± 0.2	1.1 ± 0.2	42.2 ± 1.1	1.7 ± 0.1
A15–Nb_3_X	8.1–31.1	3.1–4.5	10.3–21.0	1.2–2.7	0.6–2.2	38.1–49.7	9.2–14.7
	16.8 ± 0.8	3.9 ± 0.3	18.4 ± 1.9	2.2 ± 0.5	1.1 ± 0.5	45.7 ± 0.9	11.9 ± 0.7
Sn-rich A15–Nb_3_X	14.7–31.8	3.3–7.4	1.2–12.7	0.1–0.4	1.3–2.8	33.8–47.0	17.2–28.2
	23.0 ± 0.5	5.7 ± 0.7	5.1 ± 2.1	0.1	1.9 ± 0.4	42.9 ± 0.9	21.3 ± 0.9
Ti oxide (see text)	16.6–19.6	1.2–4.8	44.1–53.3	4.0–6.2	12.7–19.0	5.6–11.9	0.6–3.0
	17.7 ± 0.8	2.5 ± 0.8	49.6 ± 1.1	4.7 ± 0.5	15.3 ± 0.9	8.4 ± 0.8	1.8 ± 0.9
FeNb_4_Si	5.0–13.4	12.6–18.2	19.0–25.0	9.5–14.8	9.8–15.1	29.4–34.9	-
	8 ± 0.9	13.8 ± 0.8	21.0 ± 1.1	12.9 ± 0.7	11.8 ± 1.1	32.5 ± 1.3	-
Fe_7_Nb_6_	6.1–14.6	7.1–8.2	29.4–39.0	6.5–16.5	17.8–18.9	14.1–20.9	-
	10.8 ± 0.8	7.6 ± 0.2	34.2 ± 1.2	11.5 ± 1.3	18.4 ± 0.1	17.5 ± 0.5	-
Nb_3_Si m’	7.2–8.3	26.7–28.5	19.5–20.4	1.6–2.4	2.3–3.6	37.1–38.8	1.4–2.1
	7.7 ± 0.3	27.6 ± 0.5	20.0 ± 0.1	2.0 ± 0.2	2.9 ± 0.3	38.0 ± 0.5	1.8 ± 0.2

**Table 7:** Macrosegregation of Si (MACSi) in as-cast Ti-containing Nb–18Si-based RM(Nb)ICs with/without Fe and/or Sn addition.

Nominal Composition (at.%)	Alloy	MACSi (at.%)	Reference
Nb–18Si–24Ti–5Cr–5Sn	ZX4	7.3	[28]
Nb–18Si–24Ti–5Cr–5Fe–5Sn	NV5	4	[22]
Nb–18Si–24Ti–5Cr–5Fe	NV3	2.6	[22]
Nb–30Ti–10Si–5Cr–5Sn–3Fe–2Al–2Hf	NV2	2.5	This work
Nb–18Si–24Ti–5Cr	KZ4	1.9	[15]

**Table 8:** Nb_ss_, Ti-rich Nb_ss_ in as-cast (AC) and stable Nb_ss_ in heat-treated (HT) Ti-containing Nb–18Si-based RM(Nb)ICs with the solute additions used in NV2.

Nominal Composition (at.%)	Alloy	AC		HT	Reference
		Nb_ss_	Ti-Rich Nb_ss_	Nb_ss_	
Nb–18Si + 24Ti	KZ3	X	X	X	[15]
Nb–18Si +24Ti + 5Cr	KZ4	X	X	X	[15]
Nb–18Si +24Ti + 5Al	KZ7	X	X	X	[15]
Nb–18Si +24Ti + 5Al + 5Cr	KZ5	X	X	X	[15]
Nb–18Si +24Ti + 5Cr + 2Sn	ZX3	X	X	X	[27]
Nb–18Si +24Ti + 5Al + 2Sn	ZX5	X	X	X	[27]
Nb–18Si +24Ti + 5Al + 5Cr + 2Sn	ZX7	X	X	X	[27]
Nb–18Si +24Ti + 5Cr + 5Sn	ZX4	X	-	X	[28]
Nb–18Si +24Ti + 5Al + 5Sn	ZX6	X	-	-	[28]
Nb–18Si +24Ti + 5Al + 5Cr + 5Sn	ZX8	-	-	X	[28]
Nb–18Si +24Ti + 5Al + 5Sn + 5Hf	EZ5	X	-	-	[30]
Nb–18Si +24Ti + 5Cr + 5Sn + 5Hf	EZ6	X	X	X	[30]
Nb–18Si +24Ti + 5Al + 5Cr + 5Sn + 5Hf	EZ8	X	-	-	[30]
Nb–18Si +24Ti + 5Sn	NV6	X	-	X	[16]
Nb–18Si +24Ti + 5Cr + 5Fe	NV3	X	X	X	[22]
Nb–18Si +24Ti + 5Cr + 5Fe + 5Sn	NV5	X	-	-	[22]
Nb–18Si +24Ti + 5Fe + 5Sn	NV8	X	X	-	[31]

**Table 9:** Characteristic features of the microstructure of alloy NV2 oxidised at 800 °C/100 h.

Location	Phase	Characteristic Features
Oxide scale	Nb and Ti-rich oxide, Si-rich oxide, Ti-rich oxide	Thickness: non-uniform, but less severe non-uniformity compared with the alloy NV1 [32]
		Cracks still present, but noticeably less compared with NV1
		Small Sn-rich areas in scale
Diffusion zone (up to 50 μm below scale)	Nb_ss_, Nb_5_Si_3_, Nb_3_Sn, Sn-rich Nb_3_Sn, π phase	Thicker compared with the alloy NV1 and almost continuous layer of Nb_3_Sn was formed at the interface with the scale
		Cracks parallel to the oxide scale surface formed in Nb_5_Si_3_ grains
		Severely oxidised solid solution exhibiting “pitting”
		No “pitting” in Nb_3_Sn grains
Bulk (from 50 to 300 μm below scale)	Nb_ss_, Nb_5_Si_3_, Nb_3_Sn, π phase, Ti oxide	“Pitting” of Nb_ss_ almost eliminated
		No cracking of Nb_5_Si_3_ grains
		Lamellar microstructure seen in the eutectic in NV2–AC still present and intact
		Formation of Ti oxide(s) that were randomly distributed in the microstructure. Ti oxide formed mainly around the π phase and its interface with Nb_ss_, from which sometimes a lamellar microstructure of solid solution and Nb_5_Si_3_ silicide was formed

**Table 10:** Characteristic features of the microstructure of alloy NV5 oxidised at 800 °C/100 h.

Location	Phase	Characteristic Features
Oxide scale	Nb and Ti-rich oxide, Si-rich oxide, Ti-rich oxide and mixed oxide	Thickness: thinner than NV2 but still non-uniform and non-uniformity the same with NV2
		Cracking more severe than in NV2
		Small Sn-rich areas in scale
Subscale	Sn-rich Nb_3_Sn	Cracks parallel to oxide scale surface formed in Nb_5_Si_3_ grains
		Sn-rich Nb_3_Sn formed at the interface of alloy with scale. This A15 compound exhibited severe “pitting”
		Ti oxide formed in the few areas where solid solution grains were present (note that the volume fraction of Nb_ss_ in NV5–AC was about 2 at%)
Bulk (up to 300 μm below scale)	Nb_ss_, Nb_5_Si_3_, Nb_3_Sn, Nb_3_Si m’, FeNb_4_Si (τ_4_), Fe_7_Nb_6_ and Ti oxide	Volume fraction of Ti oxide increased compared with NV5–AC. Some grains of the Ti oxide were large in size
		Ti oxide was not formed near τ_4_ and Fe_7_Nb_6_
		Microstructure architecture similar to that in NV5–AC

**Table 11:** Complex concentrated or high entropy phases in the AC or HT alloy NV2 and in alloys NV1, NV2, NV5, ZX5, ZX7 and ZX8 after isothermal oxidation at 800 °C for 100 h and alloys ZX3, ZX4, ZX5 and ZX7 after isothermal oxidation at 1200 °C for 100 h.

Phase	CC	HE	Alloy	Condition or Area	Data in
Nb_5_Si_3_	X	-	NV2	AC, HT1	Table 3
Ti-lean Nb_5_Si_3_	X	-		AC	
Hf-rich Nb_5_Si_3_	X	-		AC, HT2	
Fe_7_Nb_6_ (μ phase)	X	-		AC	
Nb_3_Si	X	-		AC, HT1, HT2	
Nb_ss_ + Nb_5_Si_3_ eutectic	X	-		AC	
Si-poor Nb_5_Si_3_	X	-		HT1	
π phase	X	-		HT1, HT2	
Nb_3_Si-m’	X	-		HT1, HT2	
				**Oxidised at 800 °C**	
Nb_ss_	X	-	NV2	DZ	Table 5
Nb_5_Si_3_	X	-		DZ, Bulk	
π phase	X	-		DZ, Bulk	
Nb_ss_	X	-	NV5	Bulk	Table 6
FeNb_4_Si	-	X			
Fe_7_Nb_6_	-	X			
Nb_3_Si-m’	X	-			
Nb_5_Si_3_	X	-	NV1	Bulk	[32]
Lamellar Nb_ss_ + Nb_5_Si_3_	X	-			
Nb_ss_	X	-			
Ti-rich Nb_ss_	X	-			
Nb_ss_	X	-	ZX5, ZX7	DZ	[27]
Nb_5_Si_3_	X	-			
Lamellar Nb_ss_ + Nb_5_Si_3_	X		ZX8	DZ	[28]
				**Oxidised at 1200 °C**	
NbSn_2_	X	-	ZX3, ZX4	Sn-rich area below the scale	[27,28]
Nb_5_Si_3_	X	-	ZX5		[27]
C14–NbCr_2_	X		ZX7	Bulk	[27]

## Data Availability

All the data for this work is given in the paper, other data cannot be made available to the public.
